# Identification of Prognostic Markers for Head and NeckSquamous Cell Carcinoma Based on Glycolysis-Related Genes

**DOI:** 10.1155/2022/2762595

**Published:** 2022-07-07

**Authors:** Xiaobin Ren, Lin Jing Song, Mingzhu Zhang, Wanli Zhang, Ken Lin, Hongbing He

**Affiliations:** ^1^Department of Periodontology, The Affiliated Stomatology Hospital of Kunming Medical University, Kunming, Yunnan 650031, China; ^2^Department of Oncology, Yan'an Hospital, Kunming Medical University, Kunming, Yunnan 650000, China; ^3^Department of Otolaryngology, Kunming Children's Hospital, Kunming, Yunnan 650034, China

## Abstract

Head and neck squamous cell carcinomas (HNSCCs) comprise a heterogeneous group of tumors. Many patients respond differently to treatment and prognosis due to molecular heterogeneity. There is an urgent need to identify novel biomarkers to predict the prognosis of patients with HNSCC. Glycolysis has an important influence on the progress of HNSCC. Therefore, we investigated the prognostic significance of glycolysis-related genes in HNSCC. Our results showed that *ELF3, AURKA*, and *ADH7* of 20 glycolysis-related DEGs were significantly related to survival and were used to construct the risk signature. The risk score showed high accuracy in distinguishing the overall survival (OS) of HNSCC. The Kaplan–Meier curves demonstrated that the risk score was associated with an unfavorable prognosis in patients with female sex, male sex, grade 3, T1/2 stage, N^+^ stage, N2 stage, M0 stage, and clinical stage III/IV. Independent prognostic analysis showed that clinical stage and risk score were strongly associated with OS. Moreover, the risk score had higher accuracy in predicting 1-, 3-, and 5-year survival. *AURKA* and *ADH7* were only significantly related to M1 macrophages and neutrophils, respectively, while *ELF3* was significantly correlated with M2 macrophages and monocytes (all *p* < 0.05).The ceRNA network demonstrated that miR-335-5p and miR-9-5p may play core roles in the regulation of these three genes in HNSCC. The risk score constructed based on three glycolysis-related genes showed high accuracy in predicting the prognosis and clinicopathological characteristics of HNSCC.

## 1. Introduction

Head and neck cancers (HNCs) are a group of heterogeneous diseases with multifocal and multicellular origins, which occur in the nasal sinuses, nasal cavity, oral cavity, pharynx, throat, salivary glands, thyroid gland, etc. Squamous cell carcinomas comprise 90% of cases and are called head and neck squamous cell carcinoma (HNSCC) [1]. HNSCC, as the most common cancer type of the head and neck, is the sixth most common cancer worldwide, accounting for almost 5% of all malignant tumors [2]. Smoking, drinking, and human papillomavirus are risk factors for HNC and are related to the pathogenesis of HNSCC [3]. Although surgeries combined with radiotherapy, chemotherapy, and targeted therapy have been considered the main treatment methods in recent decades, less than 50% of patients have been cured. Moreover, the incidence of local recurrence in patients with HNSCC is 15–50% [4]. Although TNM staging is an important clinical prognostic factor to guide treatment choice, many HNSCC patients with the same TNM staging respond differently to radiotherapy, chemotherapy, and immunotherapy due to molecular heterogeneity, which greatly affects clinical efficacy and side effects [5]. Therefore, there is an urgent need to identify novel biomarkers to predict the prognosis of patients with HNSCC.

Aerobic glycolysis is one of the most important metabolic characteristics of tumor cells. It can not only provide energy for tumor cells quickly (various intermediate metabolites produced in the process are the necessary precursors for other metabolic pathways, which can provide energy for tumor cells) but also provide raw materials for the synthesis of various biological macromolecules. More importantly, high-throughput glycolysis of tumor cells produces a high-lactate, low-glucose metabolic environment that can help maintain an immunosuppressive tumor microenvironment. Drug therapy targeting glycolytic genes can inhibit tumor progression and increase sensitivity to other therapeutic agents [6]. The expression levels of the three key glycolytic enzymes, namely, hexokinase, 6-phosphofructokinase, and pyruvate kinase, are significantly increased in oral precancerous and oral squamous cell carcinoma tissues [7]. Moreover, the expression of hexokinase 2/pyruvate kinase muscle isozyme M2 (HK2/PKM2) in tongue squamous cell carcinoma tissue is significantly increased and is significantly related to tumor staging, clinical staging, and lymph node metastasis and can be used as an independent factor to predict disease prognosis. HK2/PKM2 knock-down significantly inhibited the growth of tongue squamous cell carcinoma, transplanted tumors, and lung metastasis in mice [8]. These results demonstrated the important role of glycolytic enzymes in the occurrence and development of oral cancer; thus, identifying reliable prognostic factors in patients with HNSCC based on glycolysis for accurate prognosis prediction and screening for high-risk cases are important for doctors to develop individualized treatment plans.

In recent years, the unique metabolic mode of tumor cells has attracted great attention and is expected to be an important drug target for targeted therapy [7]. Metabolic changes in the tumor microenvironment affect not only the biological characteristics of tumor cell proliferation and migration but also cause infiltration, distribution, and function changes in tumor immune cells [8]. Moreover, the Treg transcription factor Foxp3 reprograms Treg metabolism by inhibiting Myc signal transduction and glycolysis, and enhancing oxidative phosphorylation (OXPHOS) and nicotinamide adenine dinucleotide (NADH) oxidation, thereby providing Tregs the metabolic advantage in the low-glucose and high-lactic acid tumor microenvironment, thereby resisting lactate-mediated T cell function and growth inhibition [8]. However, activated T cells require large amounts of glucose, amino acids, and fatty acids and adjust their metabolic pathways to increase glycolysis and glutamate decomposition activities [9]. Activated neutrophils and M1 macrophages also rely mainly on the glycolytic pathway for energy supply [10, 11]. However, there is a lack of systematic exploration of the relationship between glucose metabolism and tumor immunity in HNSCC.

In the present study, three glycolysis genes (*ELF3, AURKA*, and *ADH7*) were significantly related to survival and were used to construct a risk signature that showed high accuracy in predicting the overall survival (OS) of HNSCC. The risk score was associated with an unfavorable prognosis in the patients with female sex, male sex, grade 3, T1/2 stage, N^+^ stage, N2 stage, M0 stage, and clinical stage III/IV. The risk score was also an independent prognostic factor for predicting the OS of patients with HNSCC. In addition, *AURKA* and *ADH7* were significantly related to M1 macrophages and neutrophils, respectively, while *ELF3* was significantly correlated with M2 macrophages and monocytes. The competing endogenous RNA (ceRNA) network demonstrated that miR-335-5p and miR-9-5p may play a core role in the regulation of these three genes in the development of HNSCC.

## 2. Materials and Methods

### 2.1. Data Source

The transcriptome and corresponding clinical data from HNSCC (500 cases) and normal (44 cases) samples were downloaded from The Cancer Genome Atlas (TCGA) database. Moreover, the transcriptome and clinical information for HNSCC and normal samples were obtained from the GSE41613 dataset in the GEO database and were considered an independent external validation set for the prognostic signature. The 288 glycolysis-related genes were obtained from the MSigDB database (https://www.gsea-msigdb.org/gsea/msigdb).

### 2.2. Identification of DEGs and Glycolysis-Related DEGs

Based on a threshold value of |log_2_ fold change (FC)| > 1 and a *p* value of <0.05, the DEGs were screened between the tumor and normal samples using the “limma” R package (v3.42.2). The DEGs were visualized by the volcano plot, and the top 50 DEGs were visualized as a heatmap. The glycolysis-related genes were intersected with the DEGs using Venn analysis (https://bioinformatics.psb.ugent.be/webtools/Venn/).

### 2.3. Univariate and Multivariate Cox Regression Analyses

The 500 HNSCC samples were divided into training (350 samples) and testing (150 samples) sets in a 7 : 3 ratio. The clinical characteristics of the HNSCC samples in the training and testing sets are reviewed in Table S1. The expression data of 20 glycolysis-related DEGs in the training set were subjected to univariate Cox regression analysis to determine those that were significantly associated with patient survival based on a *p* value of <0.2.

The genes identified in the univariate Cox regression analysis were included in the multivariate Cox regression and stepwise analyses to further filter the genes, ultimately obtaining the three genes related to prognosis, which were used to construct the risk signature. The risk score was calculated using the R package “survival” (v3.2-7) based on the linear model and predict.coxph function [12]. The risk score formula was as follows:

(1) $$\begin{array}{c} \mathrm{Risk}\;score=\frac{e^{sum\left(each\;gene's\;expression\;levels\times correspon\;di\;ng\;coefficient\right)}}{e^{sum\left(each\;gene's\;mean\;expression\;levels\times correspon\;di\;ng\;coefficient\right)}}. \end{array}$$

According to the median risk score, the 350 patients in the training set were classified into high-risk (>median) and low-risk (<median) groups. The OS and receiver operating characteristic (ROC) curve analyses were plotted respectively using the R packages “survminer” (v0.4.6) and “survivalROC” (v1.0.3) for the high- and low-risk groups in the training set. The expression levels of the three genes were visualized on a heatmap using the R package “pheatmap” (v1.0.12). The risk score system of the three genes was constructed in the training set and evaluated using the testing set and the GSE41613 dataset.

The correlations of the risk score and clinicopathological characteristics (sex, grade, clinical stage, and TNM stage) were evaluated by Kaplan–Meir survival curves using the R package “survival” (v3.2-7).

The clinicopathological characteristics were included in the univariate and multivariate Cox regression analyses to confirm the independent prognostic factors based on *p* < 0.05. A nomogram was constructed using the R package “rms” (v5.1-4) to predict the survival rates at 1, 3, and 5 years and to assess its effectiveness by calibration curves [13].

### 2.4. Distributions of the Immune Cells and Their Correlation with the Genes in the Risk Signature

The distributions of immune cells between the high- and low-risk groups were analyzed using the CIBERSORT algorithm (v1.0.3). The differential immune cells (*p* < 0.05) were selected and their correlations with the three genes in the risk signature are shown in a correlation heat map.

### 2.5. Construction of the ceRNA Network

The three micro RNAs (miRNAs) regulated by these three genes were obtained from the miRTarBase database (https://mirtarbase.cuhk.edu.cn/php/index.php). The 175 long noncoding RNAs (lncRNAs) interacting with the miRNAs were downloaded from the starBase database (https://starbase.sysu.edu.cn/). A ceRNA network was constructed and optimized using Cytoscape software (v3.7.2) using three genes, three miRNAs, and 175 lncRNAs.

### 2.6. Statistical Analyses

We used the R package “edgeR” to identify differentially expressed lncRNAs in the HNSCC and normal samples. Wilcoxon tests were used to compare the fractions of immune cells between HNSCC and normal samples in the CIBERSORT analysis. Pearson's correlation analysis was used to analyze the correlation between the three genes and differential immune cells. *p* < 0.05 was considered statistically significant.

## 3. Results

### 3.1. Identification of Glycolysis-Related DEGs

**Figure 1:**
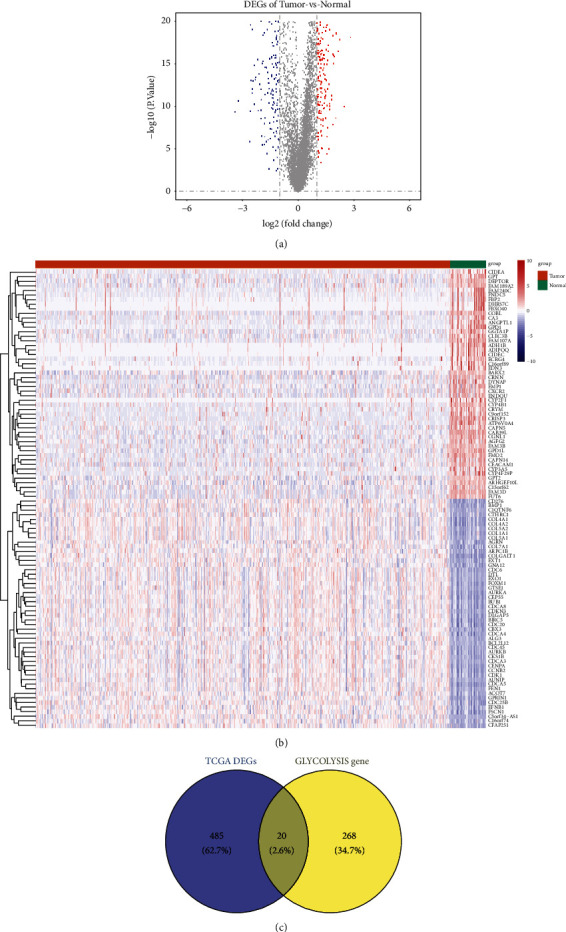
(a) Volcano plot showing the distribution of DEGs. (b) Heatmap showing the top 50 DEGs. (c) Venn diagram of DEGs common to the two GEO datasets. DEGs: differentially expressed genes; GEO: Gene Expression Omnibus.

A total of 505 DEGs (Table S2) between the HNSCC and normal samples were identified, including 293 upregulated genes and 212 downregulated genes. The distributions of DEGs were visualized in a volcano plot (Figure 1(a)), and the top 50 DEGs showed obvious differences between the HNSCC and normal samples (Figure 1(b)). Among 288 glycolysis-related genes (Table S3), 20 overlapped with the DEGs (Figure 1(c)) and were included in the follow-up analyses.

### 3.2. Construction and Verification of the Prognostic Risk Signature

**Figure 2:**
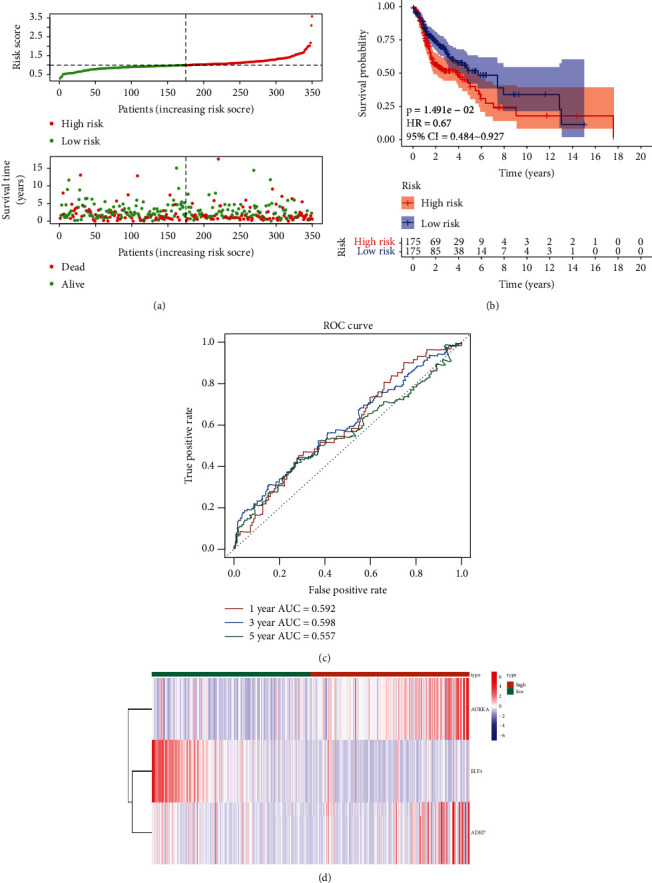
(a) Relationships between risk score and the number of deaths. (b) Overall survival of high-and low-risk patients. (c) ROC curve analysis of the predictive efficiency of the risk signature. (d) Heatmap showing AURKA, ADH7, and ELF3 expression.

To construct the glycolysis-related DEG-based prognostic signature, we performed univariate and multivariate Cox regression analyses in the training set. Univariate Cox regression analysis selected ELF3, AURKA, and ADH7 from 20 genes based on *p* < 0.2 (Table 1), and they were further included in the multivariate Cox regression analysis to determine the best characterized genes. The results are shown in Table 2, and ELF3, AURKA, and ADH7 were considered to be the optimal variables for the construction of the prognostic signature. The risk score was calculated using the aforementioned formula. The 350 samples in the training set were then divided into high-risk and low-risk groups according to the median of the risk score. The number of patients who died increased significantly as the risk score increased in the training set (Figure 2(a)). In addition, the OS differed significantly (*p*=1.491*e* − 02) between the high- and low-risk groups. The high-risk cohort was also correlated with a poor prognosis in the training set (Figure 2(b)). Subsequently, ROC curve analysis of the prognostic risk score was performed at 1, 3, and 5 years to assess the predictive efficiency of the risk score. The results are shown in Figure 2(c), and the area under the curve (AUC) of the risk score in the training set was 0.592, 0.598, and 0.557 for patients with 1, 3, and 5-year OS, respectively. The expressions of *AURKA* and *ADH7* were upregulated, while *ELF3* was downregulated in the high-risk group compared to those in the low-risk group in the training set (Figure 2(d)).

**Figure 3:**
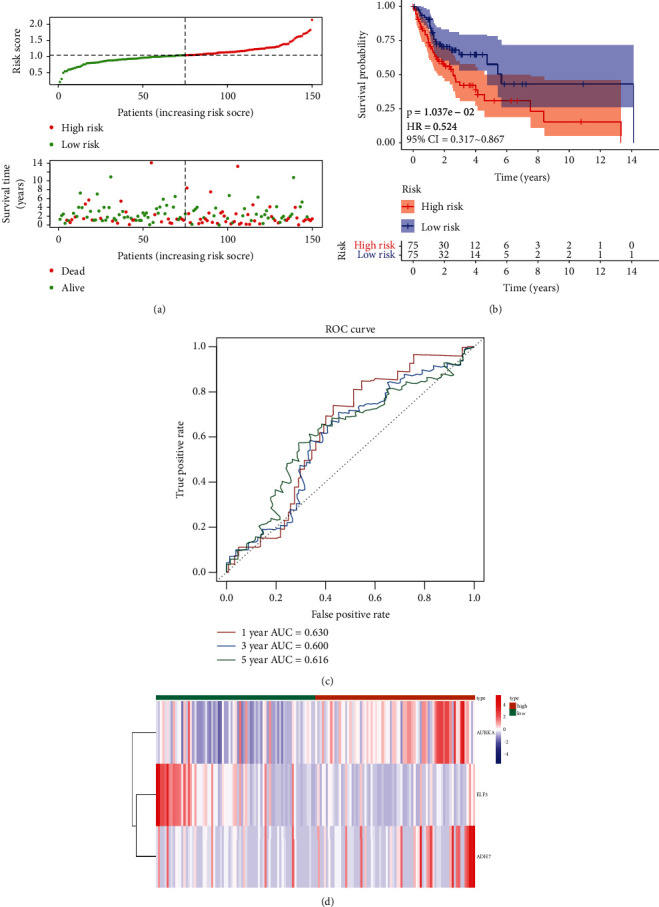
Validation set evaluating the risk score system for the three genes on the testing set. (a) Relationships between the risk score and the number of deaths. (b) Overall survival of high- and low-risk patients. (c) ROC curve analysis of the predictive efficiency of the risk signature. (d) Heatmap showing AURKA, ADH7, and ELF3 expression.

**Figure 4:**
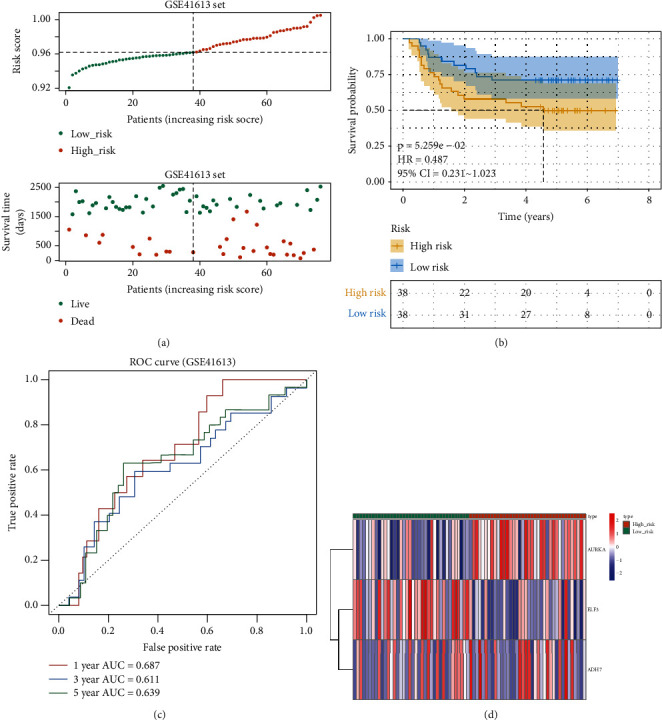
Validation set evaluating the risk score system for the three genes on the external validation set. (a) Relationships between the risk score and the number of deaths. (b) Overall survival of high- and. low-risk patients. (c) ROC curve analysis of the predictive efficiency of the risk signature. (d) Heatmap showing AURKA, ADH7, and ELF3 expression.

To demonstrate the general applicability of the risk score, we performed the same analysis in both the testing set (Figure 3) and the external validation set (Figure 4). The results showed that the risk score was able to significantly differentiate the clinical outcomes of patients (all *p* < 0.05). Meanwhile, relatively higher predictive accuracy was observed in both the testing set and the external validation set. In the testing set, the AUCs for the risk score were 0.630, 0.600, and 0.616 for OS at 1, 3, and 5 years, respectively, and 0.687, 0.611, and 0.639 in the external validation set, respectively.

### 3.3. Correlation of the Risk Score and Clinicopathological Characteristics with OS

**Figure 5:**
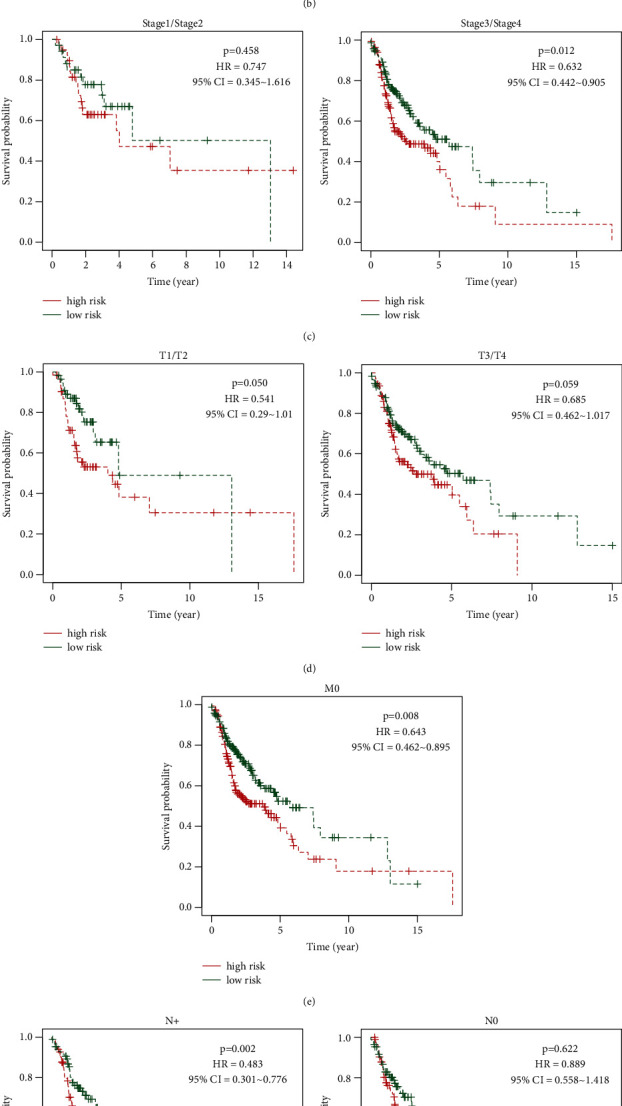
Kaplan–Meier curves to investigate the correlation of the risk score and patients' overall survival according to different patient clinicopathological characteristics.

To investigate the correlation between the risk score and clinicopathological characteristics (sex, grade, clinical stage, and TNM stage), the risk score was visualized as box plots for these clinicopathological characteristics. The results showed that the risk score did not differ significantly for these characteristics (*p* > 0.05, Figure S1). To further investigate the correlation between the risk score and patient OS, Kaplan–Meier curves were plotted in patients stratified according to clinicopathological characteristics. The results showed that the risk score was associated with an unfavorable prognosis in patients with female sex, male sex, grade 3, T1/2 stage, N^+^ stage, N2 stage, M0 stage, and clinical stage III/IV (*p* < 0.05, Figure 5), but not with grade 1/2, T3/4 stage, N0 stage, N1 stage, and clinical stage I/II (*p* > 0.05, Figure 5), suggesting that the risk scores might be associated with OS and clinicopathological characteristics.

### 3.4. Identification of Independent Prognostic Factors

**Figure 6:**
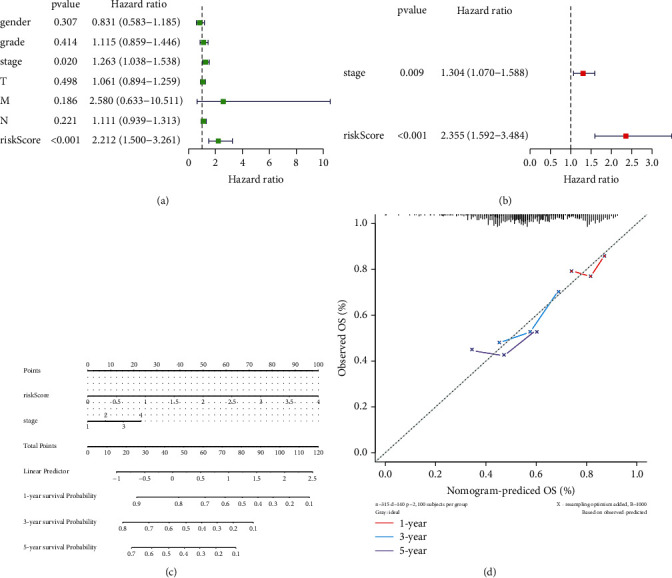
(a) The univariate analysis of the correlation on the clinical stage, risk score, and prognosis. (b) The multivariate analysis of the correlation on the clinical stage, risk score, and OS based on the abovementioned significant factors. (c) The OS of patients predicted by the nomogram model. (d) The application of calibration curves in the nomogram model.

Univariate and multivariate Cox regression analyses were performed using the TCGA dataset to explore the independent prognostic factors based on the abovementioned clinicopathological characteristics. The univariate analysis showed that the clinical stage and the risk score were significantly correlated with prognosis (*p* < 0.05, Table 3 and Figure 6(a)). In the multivariate analysis, based on the abovementioned significant factors, the clinical stage and risk score remained strongly associated with OS (clinical stage, *p*=0.009; risk score, *p* < 0.001; Table 4 and Figure 6(b)), which were considered independent prognostic factors for HNSCC. After that, we constructed a nomogram model based on these independent prognostic factors that could predict the OS of patients (Figure 6(c)). Further calibration curves showed that the nomogram model had the ability to predict the 3-year OS of patients in an approximate way to the actual observed values (Figure 6(d)), suggesting that the nomogram model may be more suitable for predicting the midterm survival probability of patients.

### 3.5. Relationships between the Genes in the Risk Signature and Immune Cells

Glycolysis-related genes were upregulated in samples of melanoma and lung cancer was poorly infiltrated by T cells. Moreover, the overexpression of glycolysis-related molecules impaired T cell killing of tumor cells, whereas the inhibition of glycolysis enhanced T cell-mediated antitumor immunity *in vitro* and *in vivo* [14]. Thus, collectively, immune cells may participate in glycolysis regulation during cancer occurrence and development.

**Figure 7:**
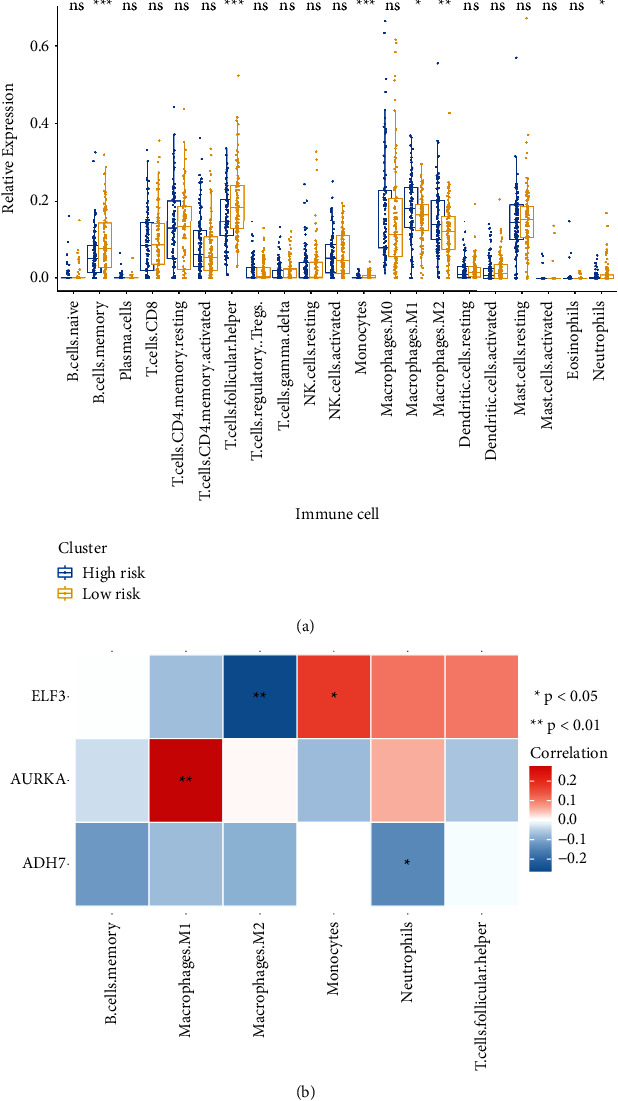
(a) CIBERSORT algorithm analyses of the relationship of the genes in the risk signature and 21 immune cells. (b) Correlation analysis of AURKA, ADH7, and ELF3 and differential immune cells. CIBERSORT: cell-type identification by estimating relative subsets of RNA transcripts.

The present study investigated the relationship between the genes in the risk signature and 21 immune cell lines using the CIBERSORT algorithm. The results showed significantly reduced fractions of B cell memory cells, T follicular helper cells, monocytes, and neutrophils in the high-risk group compared to those in the low-risk group (all*p* < 0.05). The fractions of M1 and M2 macrophages in the high-risk group were significantly higher than those in the low-risk group (*p* < 0.05, Figure 7(a)). Furthermore, the correlations of the three genes and the immune cells are shown in Figure 7(b); ELF3 showed a significant negative correlation with M2 macrophages and a significant positive correlation with monocytes; AURKA presented the highest positive relationship with M1 macrophages; ADH7 displayed a significant negative correlation with neutrophils. Taken together, the three genes related to glycolysis regulated the malignant development of HNSCC, probably by affecting the activity of immune cells, particularly macrophages, monocytes, and neutrophils. However, whether these three genes could be used as targets for immunotherapy in HNSCC requires further research.

### 3.6. Construction of the ceRNA Network Based on the Genes in the Risk Signature

**Figure 8:**
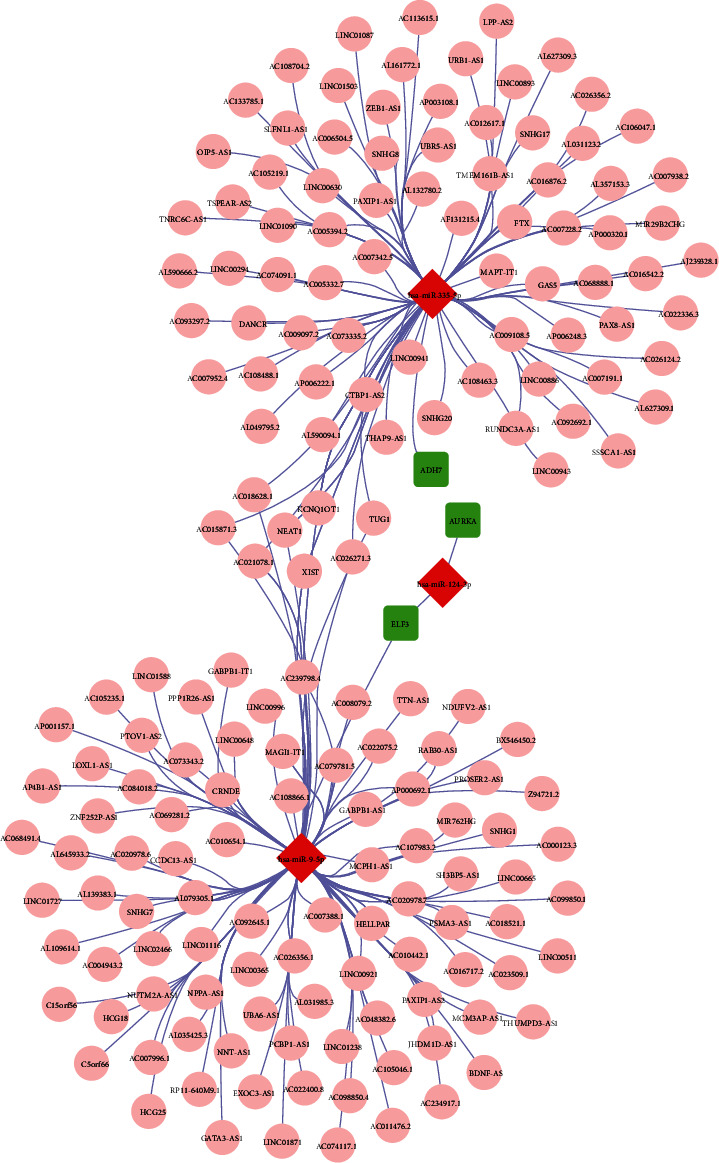
Construction of a ceRNA network based on the genes in the risk signature, ceRNA, and competitive endogenous RNA.

To investigate the coregulatory network of the genes in the risk signature, a ceRNA network was constructed using the three genes, three 3 miRNAs, and 175 lncRNAs. As shown in Figure 8, the ceRNA network was roughly divided into two clusters connected by several lncRNAs; *ADH7* was directly related to miR-335-5p, which was in a central position in a cluster and was associated with multiple lncRNAs; miR-124 was connected to both *ELF3* and *AURKA*, but not to any lncRNAs; miR-9-5p, directly connected to *ELF3* and indirectly connected to *AURKA*, was located in the center of another cluster and was related to multiple lncRNAs. These results revealed that miR-335-5p and miR-9-5p may play core roles in the regulation of these three genes in HNSCC.

## 4. Discussion

The recurrence and metastasis of head and neck tumors are the main reasons for the low OS rate of patients. Therefore, the identification of an exact tumor marker to assist in clinical diagnosis, judge patient response to treatment, and detect local recurrence and metastasis of tumors early has become a research hotspot. Cytokeratin 19 serum fragment 21-1 (Cyfra21-1), squamous cell carcinoma-associated antigen (SCCAg), tissue polypeptide-specific antigen (TPS), carcinoembryonic antigen (CEA), Fe protein, and circulating tumor cells (CTCs) are tumor markers for the diagnosis and prognosis of HNSCC [15]. However, their lack of sensitivity and specificity means that they are not considered specific tumor markers for HNSCC.

In the present study, *ELF3*, *AURKA*, and *ADH7* were significantly related to the OS of HNSCC and were used to construct a risk signature that showed high accuracy in predicting the prognosis of HNSCC. The risk scores of HNSCC patients with female sex, male sex, grade 3, T1/2, N^+^, N2, M0, and clinical stage III/IV were associated with a poor prognosis. Moreover, the risk score was an independent prognostic factor for predicting the OS of patients with HNSCC. In addition, *AURKA* and *ADH7* were significantly correlated with M1 macrophages and neutrophils, respectively, while *ELF3* was significantly correlated with M2 macrophages and monocytes. Furthermore, the ceRNA network revealed the potential roles of miR-335-5p and miR-9-5p in the regulation of these three genes in HNSCC progression.

The E26 transformation-specific (ETS) transcription factor family is located on chromosome 1q32.1 and has a conserved winged helix-turn-helix DNA-binding domain, the ETS domain, that can bind to the typical DNA sequence 50-GGA(A/T)-30 [16–18]. Members of this family can act as upstream and downstream effectors of most signaling pathways, such as MAP kinase, Erk1/2, p38, and JNK, which play important roles in cell differentiation, development, proliferation, apoptosis, tissue remodeling, and epithelial-mesenchymal transition [19–24]. *ELF3* (E74 is similar to the ETS transcription factor 3), also known as *ESE-1, EPR-1, ESX*, and *ERT*, is expressed in the nucleus and cytoplasm [25]. *ELF3* is closely associated with bladder, ovarian, biliary tract, gastric, cervical, breast, prostate, lung, liver, and colon cancer and increases cell proliferation, invasion, and migration [26, 27]. *ELF3* is highly expressed in head and neck tumors and upregulates the epidermal growth factor receptor (EGFR) and human epidermal growth factor receptor 2 (HER2) to attenuate the antiproliferative effects of EGFR/HER2 tyrosine kinase inhibitors (lapatinib and afatinib) [28].

The Aurora protein kinase family includes serine/threonine kinases that participate in many processes of cell mitosis, such as cell cycle G2/M conversion, mitotic spindle assembly, and chromosome separation [29]. The Aurora protein kinase family members include Aurora A, Aurora B, and Aurora C. The AURKA kinase is frequently amplified or overexpressed in malignant tumors such as pancreatic, prostate, gastric, breast, and colon cancers [30, 31]. Abnormal AURKA expression promotes malignant tumor occurrence and development through a variety of possible mechanisms, including promoting cell cycle processes, activating cell survival and/or antiapoptosis signal transduction, enhancing oncogene carcinogenicity, promoting the epithelial-mesenchymal transition, and dry cell transformation of cancer cells [32]. In recent years, increasing evidence has demonstrated a potential relationship between AURKA expression and tumor immunity. The initial understanding of the influence of AURKA on the immune response comes from its application in immunotherapy, in which the AURKA epitope initiated an anti-AURKA immune response, thereby killing tumor cells with high AURKA expression. Moreover, AURKA inhibition directly interfered with the immune response by inducing cell transformation, T cell activation, and immune cell infiltration [33]. Reiter et al. reported that AURKA upregulation promoted the disease stage, the occurrence of positive regional lymph nodes, and the level of distant metastasis, reducing the disease-free survival time and OS rate of patients [34]. Huang et al. [35] indicated that the risk of oral cancer increased significantly among smokers with high AURKA expression, which had a certain reference value for screening groups at high risk for oral cancer. Yang et al. [36] showed that MLN8237, an AURKA inhibitor, inhibited AURKA autophosphorylation at Thr288, leading to abnormal cell division and cell cycle to stop cell aging in the G2/M phase.

Alcoholic dehydrogenase (ADH) is a key enzyme in alcohol metabolism [37] and can be divided into five categories, including seven genotypes: ADH1a, ADH1b, ADH1c, ADH4, ADH5, ADH6, and ADH7 [38]. ADH plays an important role in the metabolism of alcohol, retinol, and other substances to oxidize ethanol to acetaldehyde. Thus, abnormal or unbalanced expression of this enzyme leads to abnormal alcohol metabolism [39]. Recently, the ADH family was reported to also play a role in the prognosis of gastric cancer, breast cancer, and nasopharyngeal carcinoma [40]. ADH7 showed promise as a prognostic biomarker and chemotherapy target for gastric adenocarcinoma, with low ADH7 expression and is related to the prognosis of patients with better histological types of adenocarcinoma and squamous cell carcinoma [41]. In addition, ADH1B + 3170A>G and ADH1C + 13044A>G single-nucleotide polymorphisms were associated with increased risks of HNSCC [42].

In conclusion, *ELF3, AURKA*, and *ADH7* were highly accurate in predicting the prognosis and clinicopathological features of patients with HNSCC, providing a new perspective for prognosis analysis and immune target therapy for HNSCC. However, this study selected few datasets and there were few tools to predict miRNA target genes. The databases used were not updated consistently and the annotation of the gene information was not perfect, which may have led to false-positive results. However, our research provides a preliminary basis and new ideas for research. Follow-up research will further focus on the functions and molecular mechanisms of these three genes miR-335-5p and miR-9-5p in the development of HNSCC. A large number of independent sample sets will also be used to verify these markers.

## 5. Conclusions

The risk score constructed based on three glycolysis-related genes showed high accuracy in predicting the prognosis and clinicopathological characteristics of HNSCC.

## Figures and Tables

**Table 1:** Univariate Cox regression analysis of 20 glycolysis-related DEGs in TCGA-training set.

Id	HR	HR.95L	HR.95H	*p* value
ADH1B	0.95880947	0.698532024	1.316067937	0.794627984
ADH1C	0.992516531	0.976875575	1.008407918	0.354002802
ADH7	1.003527529	0.998519769	1.008560403	0.167709269
AGRN	1.000417439	0.995546788	1.00531192	0.866894449
ALDH3A1	1.000187007	0.99904975	1.001325559	0.747348382
ALDH9A1	0.995777718	0.981703298	1.01005392	0.560173636
ARTN	1.004851119	0.975590593	1.034989246	0.74823725
AURKA	1.023682341	0.99982915	1.048104603	0.05168357
CAPN5	1.011790392	0.919855881	1.11291325	0.809425046
CDK1	0.993148111	0.97395806	1.012716266	0.489785082
CENPA	1.014112882	0.966000828	1.064621177	0.57199465
CHPF	1.002554489	0.997355603	1.007780476	0.336169621
CHST2	1.006487836	0.995794942	1.017295551	0.235348089
COL5A1	0.999960883	0.996736632	1.003195563	0.981060039
DDIT4	1.000084304	0.998404999	1.001766434	0.921681662
ELF3	0.991125876	0.982767465	0.999555376	0.039123533
ENO2	1.004596293	0.989552668	1.019868619	0.551376673
ENO3	1.003445564	0.997299719	1.009629283	0.27249397
EXT1	1.001927423	0.982155136	1.022097756	0.849818487
FBP2	1.006933545	0.921785842	1.099946557	0.878177364

**Table 2:** Regression coefficients for prognostic glycolysis-related DEGs.

Id	Coef	HR	HR.95L	HR.95H	*p* value
ELF3	−0.011159384	0.988902651	0.98008198	0.997802708	0.014640204
AURKA	0.021639974	1.021875816	0.998608076	1.045685698	0.065557575
ADH7	0.005465779	1.005480744	1.000514882	1.010471254	0.030484488

**Table 3:** Univariate Cox regression analysis of clinical characteristics and risk score.

Id	HR	HR.95L	HR.95H	*p* value
Gender	0.830997859	0.582541512	1.18542186	0.307044026
Grade	1.114557549	0.859062076	1.446040473	0.414259463
Stage	1.26336723	1.037522833	1.538372658	0.019989211
*T*	1.060862183	0.894131849	1.258683016	0.498248825
*M*	2.580353112	0.6334397	10.51121706	0.185900335
*N*	1.110517954	0.939063498	1.313276609	0.220518367
Risk score	2.211923156	1.500158061	3.261392365	6.15E-05

**Table 4:** Identification of independent prognostic factors of HNSCC by multivariate Cox regression analysis.

Id	HR	HR.95L	HR.95H	*p* value
Stage	1.303573262	1.069949098	1.588209432	0.008515059
Risk score	2.355354132	1.592227234	3.484234517	1.80E-05

## Data Availability

The figure data used to support the findings of this study are included within the article.

## Supplementary Materials

Supplementary Materials Table S1: the clinical characteristics of the HNSCC samples in the training and testing sets. Table S2: a total of 505 DEGs between the HNSCC and normal samples. Table S3: 288 glycolysis-related genes. Figure S1: the correlation between the risk score and clinicopathological characteristics.
