# Supplementary material for: Identification of Prognostic Markers for Head and NeckSquamous Cell Carcinoma Based on Glycolysis-Related Genes
Source: Evid Based Complement Alternat Med. 2022 Jul 7;2022:2762595. doi: 10.1155/2022/2762595 (PMC9283050; doi:10.1155/2022/2762595)
Supplement: Supplementary Materials — Table S1: the clinical characteristics of the HNSCC samples in the training and testing sets. Table S2: a total of 505 DEGs between the HNSCC and normal samples. Table S3: 288 glycolysis-related genes. Figure S1: the correlation between the risk score and clinicopathological characteristics. [file 2762595.f1.zip › 2762595.f1/Figure supplement 1.pdf]

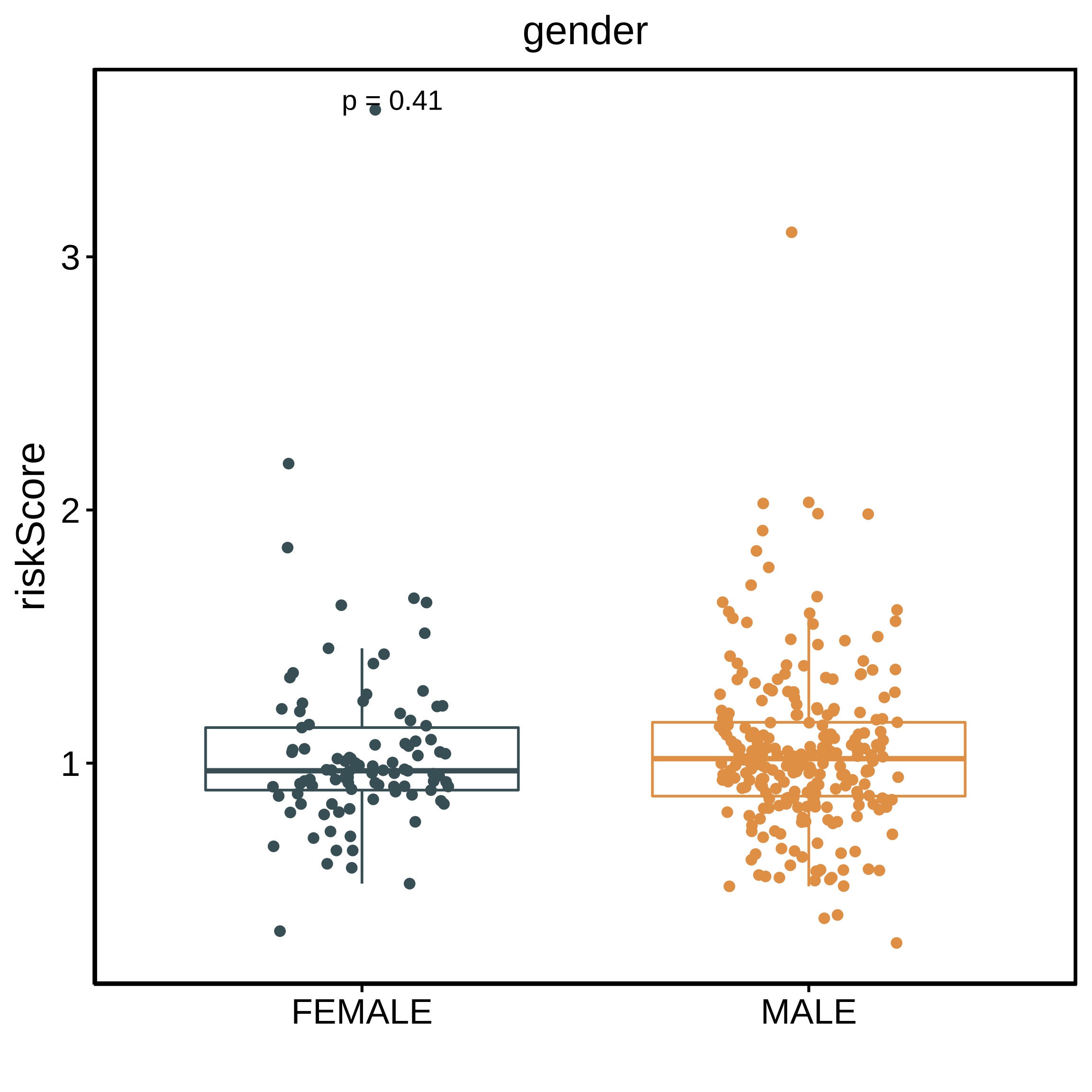
gender
p = 0.41
riskScore
3
2
1
FEMALE
MALE

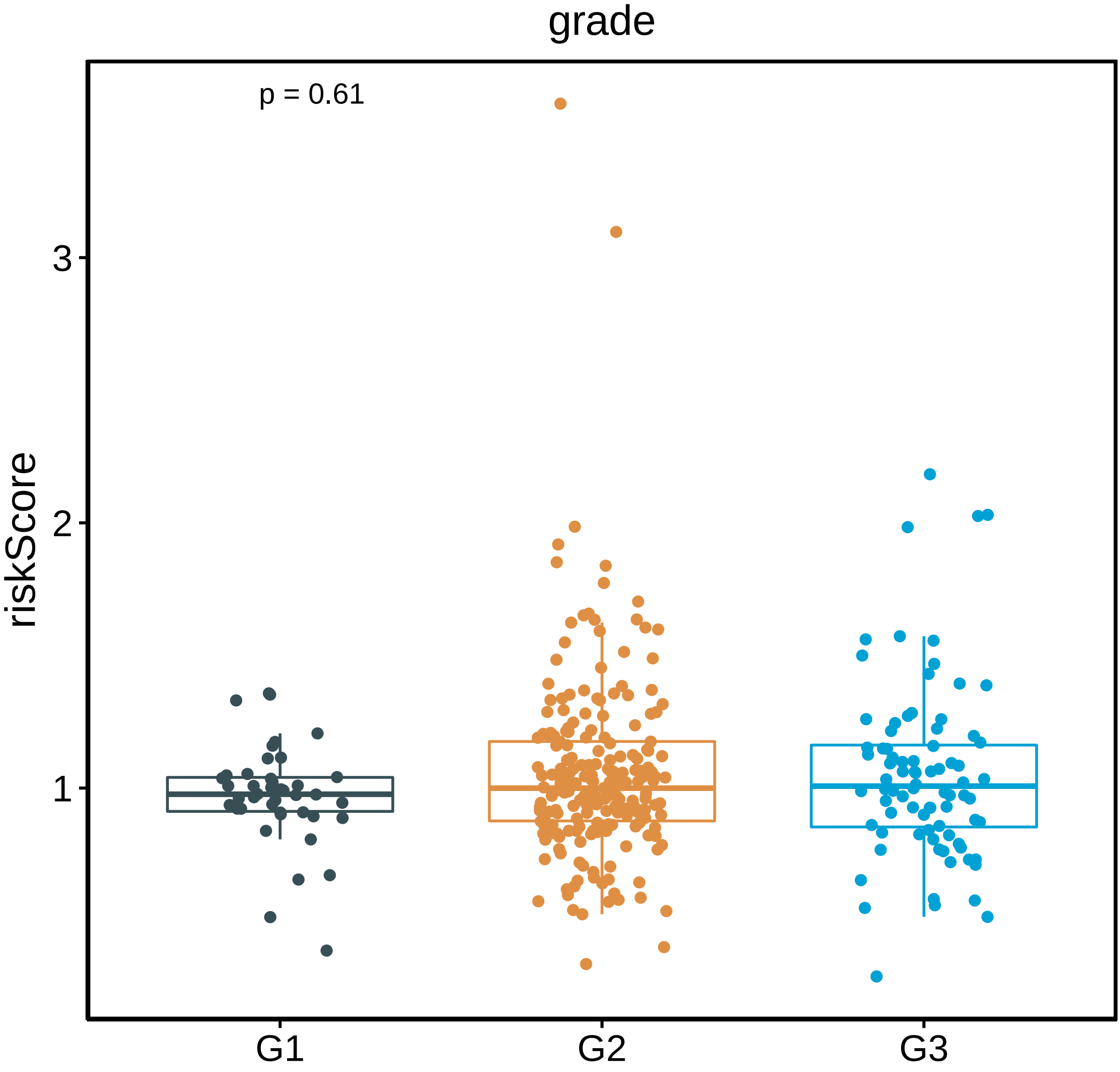
grade
p = 0.61
riskScore
3
2
1
G1
G2
G3

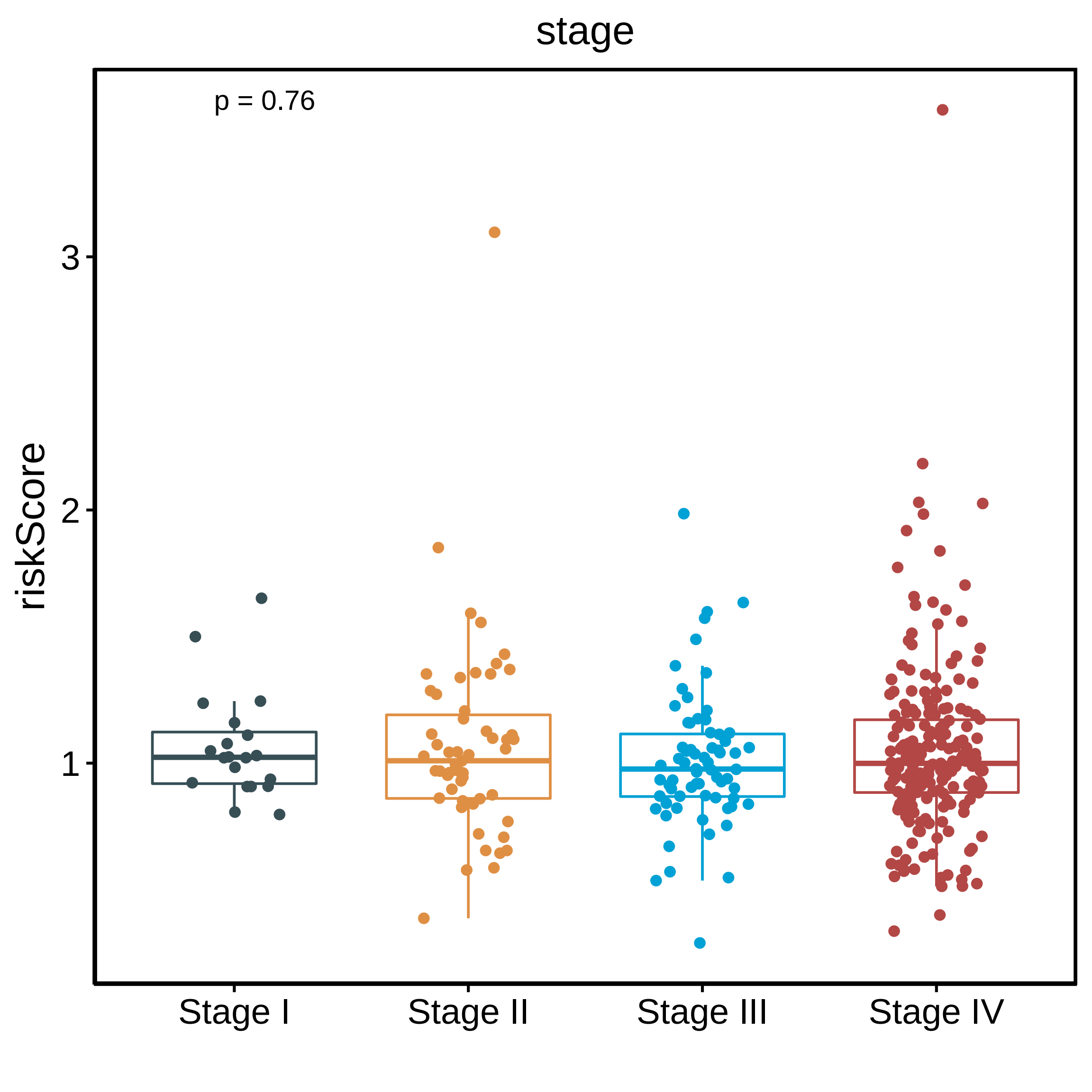

stage
p = 0.76
riskScore
3
2
1
Stage I
Stage II
Stage III
Stage IV

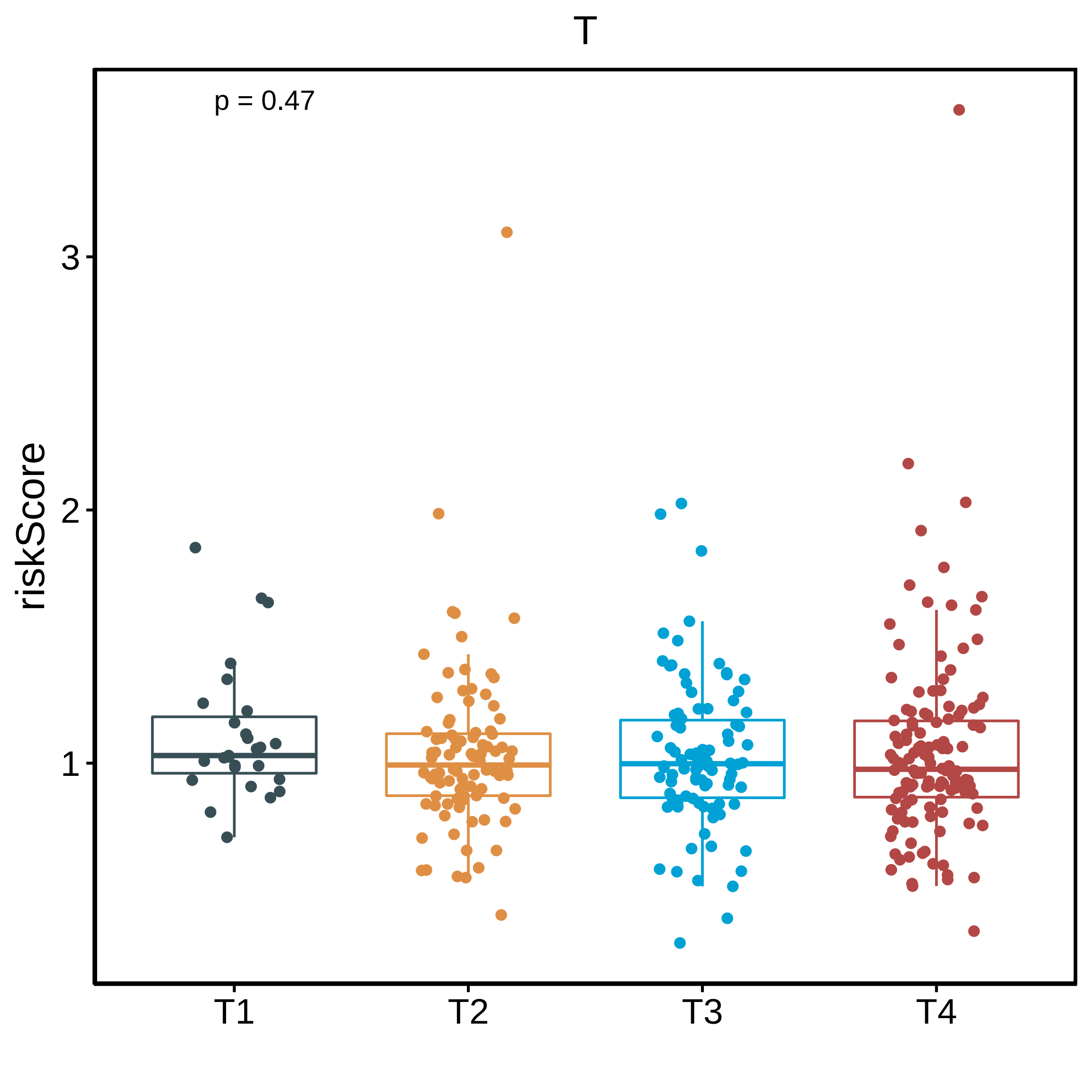
T
p = 0.47
riskScore
3
2
1
T1
T2
T3
T4

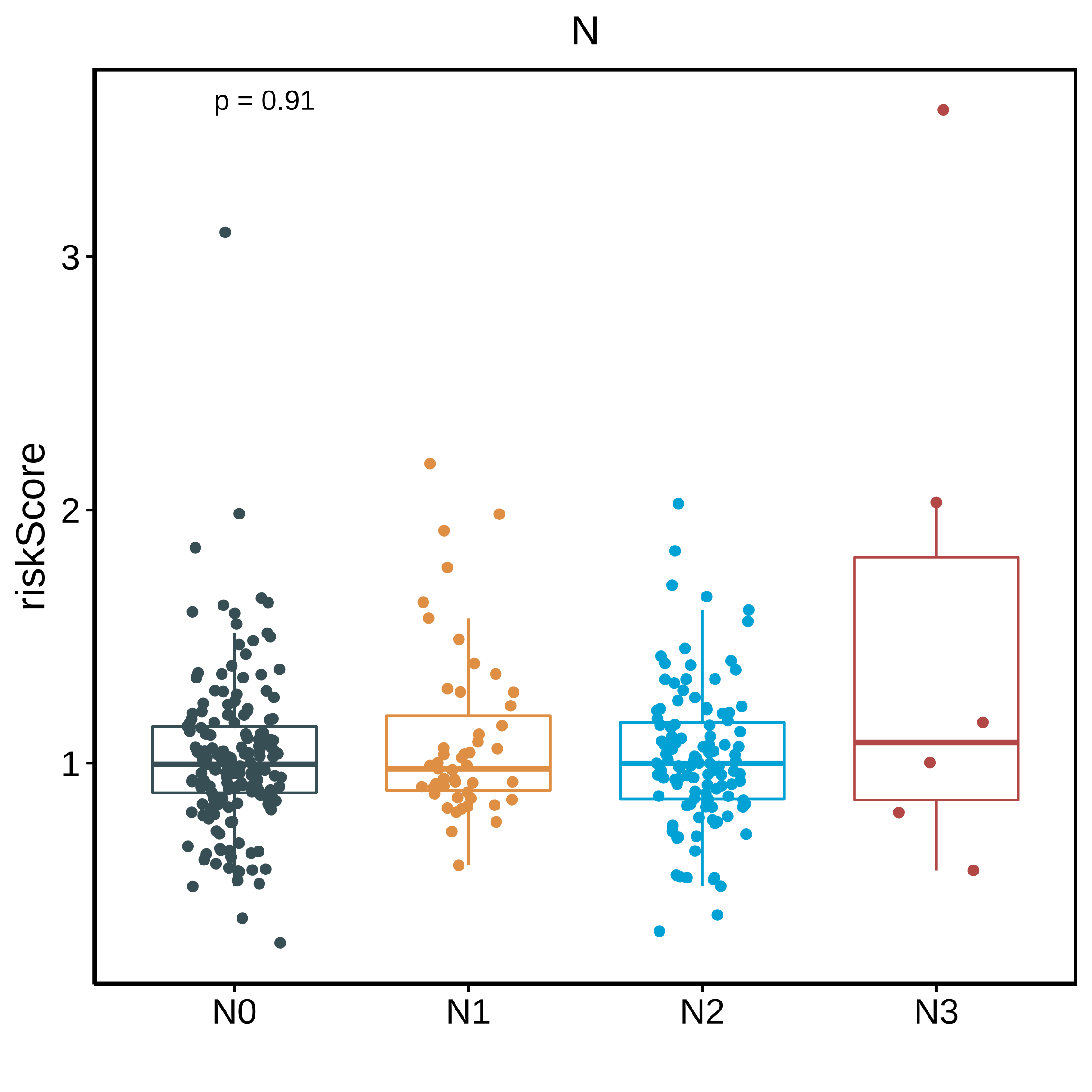
N
p = 0.91
riskScore
3
2
1
N0
N1
N2
N3

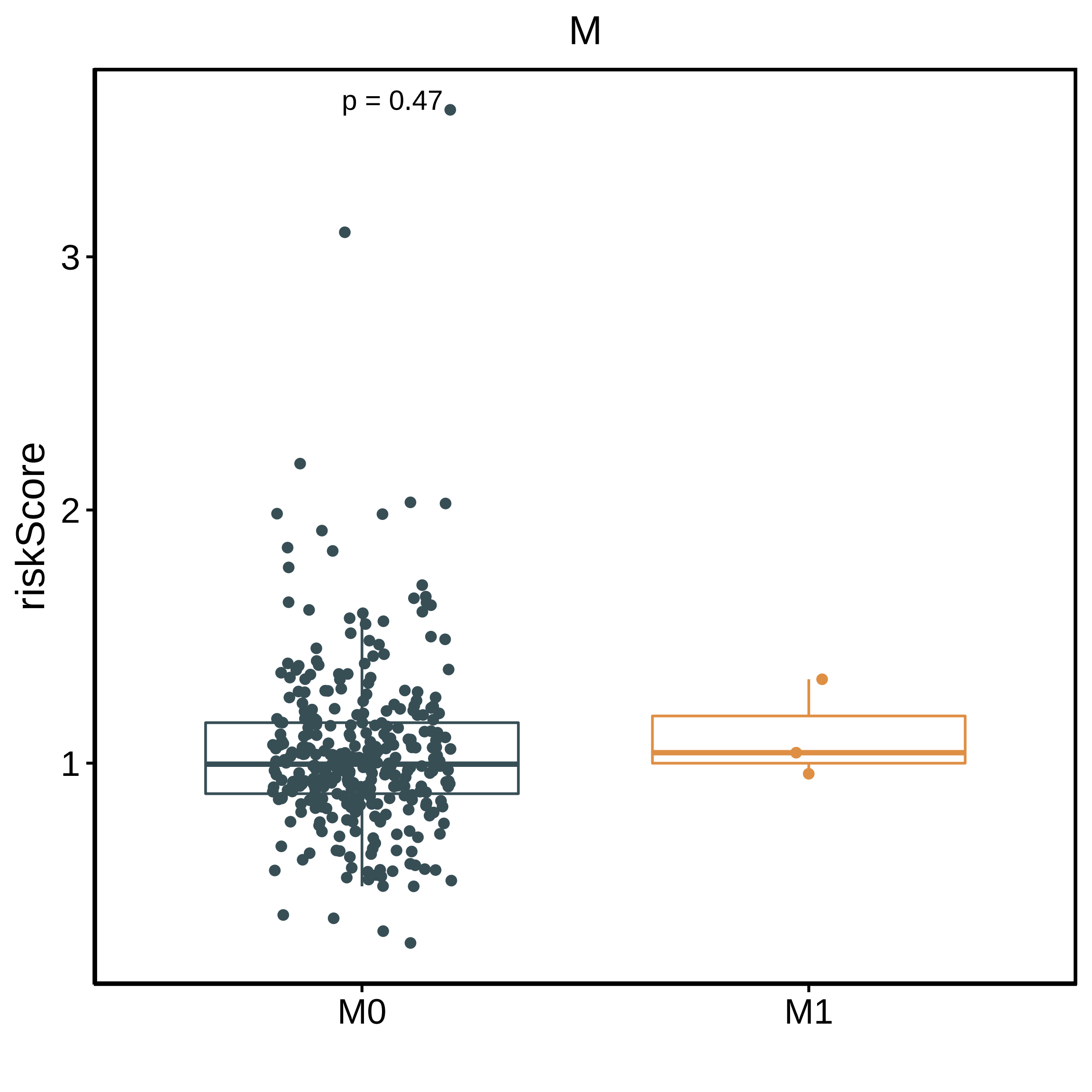
M
p = 0.47
riskScore
3
2
1
M0
M1
