# Supplementary material for: Identification of Prognostic Markers for Head and NeckSquamous Cell Carcinoma Based on Glycolysis-Related Genes
Source: Evid Based Complement Alternat Med. 2022 Jul 7;2022:2762595. doi: 10.1155/2022/2762595 (PMC9283050; doi:10.1155/2022/2762595)
Supplement: Supplementary Materials — Table S1: the clinical characteristics of the HNSCC samples in the training and testing sets. Table S2: a total of 505 DEGs between the HNSCC and normal samples. Table S3: 288 glycolysis-related genes. Figure S1: the correlation between the risk score and clinicopathological characteristics. [file 2762595.f1.zip › 2762595.f1/SUPPLEMENTARY DESCRIPTION.docx]

SUPPLEMENTARY DESCRIPTION

Table S1 The clinical characteristics of the HNSCC samples in the training and testing sets

Table S2 A total of 505 DEGs between HNSCC and normal samples

Table S3 288 glycolysis-related genes

FigureS1 The correlation between the risk score and clinicopathological characteristics
