# Supplementary material for: Identification of Prognostic Markers for Head and NeckSquamous Cell Carcinoma Based on Glycolysis-Related Genes
Source: Evid Based Complement Alternat Med. 2022 Jul 7;2022:2762595. doi: 10.1155/2022/2762595 (PMC9283050; doi:10.1155/2022/2762595)
Supplement: Supplementary Materials — Table S1: the clinical characteristics of the HNSCC samples in the training and testing sets. Table S2: a total of 505 DEGs between the HNSCC and normal samples. Table S3: 288 glycolysis-related genes. Figure S1: the correlation between the risk score and clinicopathological characteristics. [file 2762595.f1.zip › 2762595.f1/Table S2.docx]

Table S2 A total of 505 DEGs between HNSCC and normal samples

|  | logFC | AveExpr | t | P.Value | adj.P.Val | B |
| --- | --- | --- | --- | --- | --- | --- |
| CRISP3 | -4.956934205 | 1.133212852 | -21.03359052 | 2.80E-72 | 2.80E-68 | 153.7567985 |
| ADH1B | -1.660871302 | 0.284629042 | -20.36469519 | 6.53E-69 | 3.26E-65 | 146.0713788 |
| CAB39L | -1.287181442 | 1.222384069 | -19.57678077 | 5.80E-65 | 1.93E-61 | 137.0601163 |
| FAM107A | -1.802337368 | 0.725797023 | -19.41432439 | 3.75E-64 | 9.38E-61 | 135.2090434 |
| ATP6V0A4 | -2.111002884 | 0.724676321 | -19.39375989 | 4.75E-64 | 9.50E-61 | 134.9749144 |
| GPD1 | -2.090146108 | 0.489420571 | -19.23750756 | 2.86E-63 | 4.76E-60 | 133.1974044 |
| COLGALT1 | 1.641648642 | 5.099663939 | 19.19984334 | 4.40E-63 | 6.28E-60 | 132.7693294 |
| CIDEC | -1.060858266 | 0.165267692 | -17.14103742 | 5.88E-53 | 6.53E-50 | 109.6588813 |
| CDCA5 | 1.880644907 | 3.559642026 | 16.68988719 | 8.84E-51 | 8.04E-48 | 104.6892051 |
| FMO2 | -3.133569918 | 1.301890433 | -16.62218581 | 1.87E-50 | 1.56E-47 | 103.9469948 |
| GPD1L | -1.979599887 | 2.134107857 | -16.58900759 | 2.70E-50 | 2.08E-47 | 103.5836146 |
| GPRIN1 | 1.586102642 | 2.353208268 | 16.5702521 | 3.32E-50 | 2.37E-47 | 103.3783009 |
| FAM3D | -4.202109649 | 2.202770964 | -16.32830857 | 4.77E-49 | 2.98E-46 | 100.7366199 |
| CD276 | 1.929077956 | 4.497543484 | 16.20669902 | 1.81E-48 | 1.01E-45 | 99.41373736 |
| ADIPOQ | -1.250830057 | 0.174142199 | -16.11826121 | 4.78E-48 | 2.51E-45 | 98.45383274 |
| COL4A1 | 2.786867103 | 5.259742391 | 16.0886173 | 6.61E-48 | 3.30E-45 | 98.13248568 |
| COBL | -1.43724714 | 0.725751997 | -15.82448902 | 1.18E-46 | 5.35E-44 | 95.27855058 |
| CGNL1 | -1.73811804 | 0.898803285 | -15.81600193 | 1.29E-46 | 5.61E-44 | 95.18712878 |
| COL4A2 | 2.589730914 | 5.450906106 | 15.7844402 | 1.82E-46 | 7.58E-44 | 94.84730719 |
| BMP1 | 1.644111656 | 3.499464484 | 15.56372201 | 1.99E-45 | 7.64E-43 | 92.47789904 |
| BIRC5 | 1.858153375 | 4.086933018 | 15.52028466 | 3.18E-45 | 1.18E-42 | 92.01307722 |
| FSCN1 | 2.102615523 | 7.207589576 | 15.42841445 | 8.55E-45 | 3.05E-42 | 91.03161341 |
| CDC20 | 1.974108479 | 5.283180499 | 15.29545136 | 3.57E-44 | 1.23E-41 | 89.61514938 |
| C9orf152 | -1.280995534 | 0.348754663 | -15.25527812 | 5.49E-44 | 1.83E-41 | 89.18812753 |
| CEP55 | 1.877869512 | 3.736092724 | 15.23441364 | 6.87E-44 | 2.22E-41 | 88.96652366 |
| CLEC3B | -2.07128671 | 1.621745775 | -15.18709661 | 1.14E-43 | 3.56E-41 | 88.46441114 |
| EDN3 | -1.36072842 | 0.230027323 | -15.13196704 | 2.06E-43 | 6.23E-41 | 87.8801827 |
| CYP4B1 | -2.627800144 | 1.074845708 | -14.97287716 | 1.12E-42 | 3.21E-40 | 86.19907881 |
| AURKA | 1.660100263 | 3.367968783 | 14.89023518 | 2.70E-42 | 7.30E-40 | 85.32868152 |
| CENPA | 1.539311062 | 2.595940718 | 14.86279417 | 3.62E-42 | 9.52E-40 | 85.04011145 |
| CYP2F1 | -1.079883435 | 0.194494648 | -14.77164045 | 9.50E-42 | 2.38E-39 | 84.08313967 |
| COL7A1 | 2.345169285 | 4.88889476 | 14.68359053 | 2.41E-41 | 5.88E-39 | 83.16111945 |
| CDCA4 | 1.384698598 | 4.356935644 | 14.6441854 | 3.65E-41 | 8.69E-39 | 82.74924876 |
| C16orf89 | -1.260648339 | 0.3055137 | -14.49509247 | 1.75E-40 | 4.08E-38 | 81.19523332 |
| GNA12 | 1.443924686 | 3.825477917 | 14.36955741 | 6.53E-40 | 1.45E-37 | 79.89217659 |
| CCNB2 | 1.625191669 | 3.700009019 | 14.18827595 | 4.33E-39 | 9.02E-37 | 78.01942442 |
| CDC25B | 1.750585881 | 5.079577014 | 14.14481432 | 6.80E-39 | 1.36E-36 | 77.57204091 |
| AGFG2 | -1.372309423 | 2.096058474 | -14.13212113 | 7.76E-39 | 1.52E-36 | 77.44149875 |
| GPT | -1.471375249 | 0.78842193 | -14.12600994 | 8.26E-39 | 1.56E-36 | 77.37866781 |
| GGTA1P | -1.228251366 | 0.873164712 | -14.01293892 | 2.67E-38 | 4.85E-36 | 76.21841478 |
| ANGPTL1 | -1.318116711 | 0.492092906 | -13.94544285 | 5.35E-38 | 9.56E-36 | 75.52788585 |
| FOXM1 | 1.907838711 | 3.788728934 | 13.88074059 | 1.04E-37 | 1.83E-35 | 74.86740914 |
| CDC6 | 1.537650169 | 2.76435863 | 13.82554836 | 1.84E-37 | 3.17E-35 | 74.30515828 |
| EXT1 | 1.401698247 | 3.977941185 | 13.79108046 | 2.62E-37 | 4.45E-35 | 73.95456966 |
| CYP4F29P | -1.748796788 | 0.52290262 | -13.70447812 | 6.37E-37 | 1.03E-34 | 73.07554878 |
| CDC45 | 1.633965961 | 2.999022401 | 13.69023316 | 7.37E-37 | 1.17E-34 | 72.93121659 |
| DLGAP5 | 1.518905233 | 3.095951383 | 13.63879987 | 1.25E-36 | 1.95E-34 | 72.41069177 |
| CBX3 | 1.107866618 | 5.125710486 | 13.55870464 | 2.82E-36 | 4.21E-34 | 71.60199836 |
| AURKB | 1.822749985 | 3.760177962 | 13.53424563 | 3.62E-36 | 5.32E-34 | 71.35550956 |
| FUT6 | -2.346133107 | 1.153719341 | -13.5150159 | 4.40E-36 | 6.38E-34 | 71.16187338 |
| CDCA8 | 1.604653884 | 3.594403246 | 13.50319796 | 4.96E-36 | 7.09E-34 | 71.04293848 |
| ARPC1B | 1.518794551 | 5.615827345 | 13.44152649 | 9.28E-36 | 1.31E-33 | 70.42311733 |
| COL1A1 | 3.774204358 | 7.807969902 | 13.41066569 | 1.27E-35 | 1.76E-33 | 70.11348434 |
| AUNIP | 1.110188324 | 1.744679989 | 13.34058514 | 2.58E-35 | 3.53E-33 | 69.41167371 |
| AGRN | 1.760116358 | 5.34645186 | 13.28513872 | 4.51E-35 | 6.10E-33 | 68.85772561 |
| DYNAP | -2.207361568 | 0.638188384 | -13.15335301 | 1.70E-34 | 2.23E-32 | 67.54580359 |
| ECRG4 | -1.193712921 | 0.345324108 | -13.13250763 | 2.09E-34 | 2.72E-32 | 67.33890193 |
| FNDC5 | -1.680174436 | 0.574552878 | -13.1297739 | 2.15E-34 | 2.76E-32 | 67.3117807 |
| CKS1B | 1.470415766 | 3.894749907 | 13.09654582 | 3.00E-34 | 3.80E-32 | 66.98235904 |
| CFAP251 | 1.332619922 | 1.87746816 | 13.07660221 | 3.66E-34 | 4.58E-32 | 66.78484618 |
| CAPN5 | -1.562728963 | 1.471494555 | -13.04324211 | 5.11E-34 | 6.31E-32 | 66.4548109 |
| C1QTNF6 | 1.863286246 | 2.468963139 | 12.9879156 | 8.88E-34 | 1.08E-31 | 65.90842698 |
| FEN1 | 1.389367743 | 4.411293567 | 12.94245582 | 1.40E-33 | 1.66E-31 | 65.46039252 |
| ACOT7 | 1.334762813 | 4.249583943 | 12.88706676 | 2.42E-33 | 2.82E-31 | 64.91561553 |
| CDKN3 | 1.465145927 | 3.149841923 | 12.88670019 | 2.43E-33 | 2.82E-31 | 64.91201428 |
| BUB1 | 1.287566309 | 2.498599902 | 12.8173996 | 4.83E-33 | 5.55E-31 | 64.23216322 |
| C5orf34-AS1 | 1.530525694 | 1.665940268 | 12.79038496 | 6.31E-33 | 7.09E-31 | 63.96767305 |
| CRYM | -1.205785437 | 0.462779749 | -12.76305485 | 8.26E-33 | 9.18E-31 | 63.70039763 |
| FAM189A2 | -1.388239982 | 0.773470175 | -12.72758173 | 1.17E-32 | 1.29E-30 | 63.35394437 |
| GTSE1 | 1.303444345 | 2.275730763 | 12.68639988 | 1.76E-32 | 1.89E-30 | 62.95238662 |
| ALG3 | 1.193091348 | 4.382147063 | 12.68144114 | 1.85E-32 | 1.97E-30 | 62.90408208 |
| CDCA3 | 1.23137874 | 2.034032142 | 12.65323403 | 2.44E-32 | 2.55E-30 | 62.62950252 |
| EXO1 | 1.2680684 | 2.064881222 | 12.65284651 | 2.45E-32 | 2.55E-30 | 62.62573249 |
| EFNB1 | 1.552563834 | 5.75324711 | 12.61874786 | 3.42E-32 | 3.46E-30 | 62.29425019 |
| COL5A2 | 2.912462846 | 4.789603809 | 12.55189037 | 6.59E-32 | 6.46E-30 | 61.64572453 |
| COL5A1 | 2.947907256 | 4.716002468 | 12.49395133 | 1.16E-31 | 1.11E-29 | 61.0852351 |
| CTHRC1 | 2.727588217 | 4.009826698 | 12.45040881 | 1.78E-31 | 1.68E-29 | 60.66495432 |
| ARHGEF10L | -1.172080784 | 2.415725388 | -12.38685994 | 3.30E-31 | 3.02E-29 | 60.05302712 |
| DHRS7C | -1.530767968 | 0.274701602 | -12.38616089 | 3.32E-31 | 3.02E-29 | 60.04630546 |
| BCL2L12 | 1.118559087 | 3.305107898 | 12.37927649 | 3.55E-31 | 3.17E-29 | 59.98012048 |
| C16orf74 | 1.665515017 | 3.130600178 | 12.32082168 | 6.25E-31 | 5.49E-29 | 59.41897763 |
| DEPTOR | -1.931811428 | 1.795680811 | -12.30562296 | 7.25E-31 | 6.28E-29 | 59.27331954 |
| EMP1 | -2.202472174 | 5.271687397 | -12.30517046 | 7.28E-31 | 6.28E-29 | 59.26898446 |
| DTL | 1.418319222 | 2.463062638 | 12.25647162 | 1.17E-30 | 9.97E-29 | 58.80296556 |
| CDK1 | 1.451380211 | 3.647412528 | 12.21985583 | 1.66E-30 | 1.40E-28 | 58.4532606 |
| CDH24 | 1.20589198 | 2.132807024 | 12.21223107 | 1.79E-30 | 1.49E-28 | 58.38051361 |
| CIP2A | 1.24974261 | 2.107654944 | 12.16731837 | 2.75E-30 | 2.28E-28 | 57.95252892 |
| FZD2 | 1.30283018 | 1.899930074 | 12.1227935 | 4.23E-30 | 3.44E-28 | 57.52912588 |
| CGAS | 1.317638084 | 2.539650456 | 12.12081242 | 4.31E-30 | 3.47E-28 | 57.51030765 |
| CCNB1 | 1.531100786 | 4.798706886 | 12.11229952 | 4.67E-30 | 3.71E-28 | 57.42946387 |
| FAM3B | -2.659846423 | 1.278932864 | -12.08472993 | 6.09E-30 | 4.76E-28 | 57.16786912 |
| C11orf24 | 1.020156442 | 3.855937953 | 12.07521726 | 6.67E-30 | 5.17E-28 | 57.07768722 |
| CHST11 | 1.614704426 | 2.811203564 | 12.06270139 | 7.52E-30 | 5.78E-28 | 56.9590965 |
| COL12A1 | 2.552415499 | 4.194167733 | 12.00638425 | 1.29E-29 | 9.55E-28 | 56.42635494 |
| GINS1 | 1.284725383 | 2.684548781 | 11.90485386 | 3.39E-29 | 2.44E-27 | 55.46956096 |
| FAM240C | -1.377427999 | 0.53724762 | -11.87595345 | 4.46E-29 | 3.16E-27 | 55.19807707 |
| ENDOU | -2.570302689 | 1.493588428 | -11.85464488 | 5.46E-29 | 3.85E-27 | 54.99815646 |
| ERF | 1.016559171 | 4.249043072 | 11.84484716 | 5.99E-29 | 4.19E-27 | 54.90630328 |
| CENPN | 1.086884603 | 2.576429661 | 11.80535277 | 8.71E-29 | 6.00E-27 | 54.5364974 |
| AP2M1 | 1.025080524 | 6.786452622 | 11.80467638 | 8.77E-29 | 6.00E-27 | 54.53017035 |
| CCNA2 | 1.4564949 | 3.788261032 | 11.80318906 | 8.89E-29 | 6.05E-27 | 54.51625853 |
| CCM2 | 1.126259471 | 3.338506377 | 11.78810553 | 1.03E-28 | 6.93E-27 | 54.3752309 |
| CIDEA | -1.227204072 | 0.43280544 | -11.7689881 | 1.23E-28 | 8.24E-27 | 54.19664026 |
| CRNN | -5.624304531 | 3.204274763 | -11.74152272 | 1.59E-28 | 1.05E-26 | 53.94036498 |
| CKS2 | 1.622250602 | 5.736160829 | 11.73711627 | 1.66E-28 | 1.08E-26 | 53.89928201 |
| ELF4 | 1.274075324 | 3.920602285 | 11.72336124 | 1.89E-28 | 1.22E-26 | 53.77109783 |
| CAPN14 | -2.46915547 | 1.196241929 | -11.69053606 | 2.57E-28 | 1.64E-26 | 53.46555809 |
| COL27A1 | 1.405117454 | 2.202390167 | 11.68731497 | 2.65E-28 | 1.68E-26 | 53.43560335 |
| FBP2 | -1.497649458 | 0.423640047 | -11.66066391 | 3.41E-28 | 2.13E-26 | 53.18794884 |
| ATAD2 | 1.415913267 | 3.132267762 | 11.62064827 | 4.96E-28 | 3.06E-26 | 52.81673868 |
| FBXO40 | -1.058107028 | 0.200708758 | -11.61593505 | 5.18E-28 | 3.18E-26 | 52.77306622 |
| CCNF | 1.168741973 | 2.543266716 | 11.61469982 | 5.24E-28 | 3.20E-26 | 52.76162241 |
| EIF2AK2 | 1.176333861 | 3.258442765 | 11.57612763 | 7.52E-28 | 4.51E-26 | 52.40463665 |
| CXCR2 | -1.493025091 | 1.051639912 | -11.56776672 | 8.13E-28 | 4.84E-26 | 52.32735063 |
| COL4A5 | 1.711223075 | 2.943506585 | 11.50317398 | 1.49E-27 | 8.69E-26 | 51.731408 |
| FANCI | 1.23776805 | 2.872031621 | 11.48421072 | 1.77E-27 | 1.03E-25 | 51.55683353 |
| DNAJB11 | 1.034718742 | 4.129767872 | 11.42902062 | 2.96E-27 | 1.69E-25 | 51.04975332 |
| CEACAM1 | -2.110098657 | 2.164676422 | -11.42283634 | 3.14E-27 | 1.77E-25 | 50.99302557 |
| CKAP2L | 1.208877022 | 2.279987263 | 11.4056161 | 3.68E-27 | 2.07E-25 | 50.83516494 |
| AIM2 | 2.381506552 | 2.898196224 | 11.40355868 | 3.75E-27 | 2.09E-25 | 50.81631392 |
| BST2 | 2.811746144 | 7.121100127 | 11.4031012 | 3.77E-27 | 2.09E-25 | 50.81212267 |
| CDT1 | 1.44349095 | 3.258546551 | 11.39928865 | 3.90E-27 | 2.16E-25 | 50.7771967 |
| FBXL6 | 1.118831681 | 3.012376895 | 11.37894763 | 4.71E-27 | 2.59E-25 | 50.59097822 |
| DEPDC1B | 1.11692209 | 1.883914379 | 11.37393371 | 4.94E-27 | 2.70E-25 | 50.54510791 |
| CERCAM | 1.780837013 | 3.330409695 | 11.3515357 | 6.07E-27 | 3.30E-25 | 50.34034914 |
| BARX2 | -2.21552729 | 2.891762158 | -11.33712542 | 6.94E-27 | 3.75E-25 | 50.20874389 |
| C15orf62 | -1.561635921 | 1.48717126 | -11.31469673 | 8.53E-27 | 4.56E-25 | 50.00411319 |
| ADA | 1.462665452 | 3.19009135 | 11.31037436 | 8.88E-27 | 4.71E-25 | 49.96470625 |
| CA9 | 3.152487615 | 3.223019135 | 11.31004305 | 8.91E-27 | 4.71E-25 | 49.96168606 |
| CYP3A5 | -1.088632634 | 0.557471429 | -11.29221199 | 1.05E-26 | 5.50E-25 | 49.79922176 |
| ECT2 | 1.520962363 | 3.533073471 | 11.27707389 | 1.21E-26 | 6.22E-25 | 49.66141801 |
| EIF5A2 | 1.073660608 | 1.652652782 | 11.25555698 | 1.47E-26 | 7.54E-25 | 49.46574405 |
| CLIC4 | 1.259582156 | 5.231288595 | 11.22053635 | 2.03E-26 | 1.03E-24 | 49.1477631 |
| ADAMTS2 | 2.208904576 | 2.97689426 | 11.2173899 | 2.09E-26 | 1.05E-24 | 49.11922404 |
| DNMT3B | 1.171103824 | 1.571024012 | 11.18249489 | 2.88E-26 | 1.42E-24 | 48.80305159 |
| BUB1B | 1.25247758 | 2.57059527 | 11.17633098 | 3.04E-26 | 1.49E-24 | 48.74726611 |
| CA3 | -2.474137017 | 1.056427471 | -11.17419545 | 3.10E-26 | 1.51E-24 | 48.72794336 |
| ANLN | 1.53889118 | 3.720714622 | 11.14202584 | 4.17E-26 | 1.99E-24 | 48.43714414 |
| ASF1B | 1.532893907 | 3.732959103 | 11.14132786 | 4.19E-26 | 2.00E-24 | 48.43084047 |
| GPT2 | -1.444232778 | 3.030907496 | -11.1322139 | 4.56E-26 | 2.16E-24 | 48.34855278 |
| DCBLD1 | 1.482397195 | 2.631803781 | 11.12524897 | 4.86E-26 | 2.29E-24 | 48.28569656 |
| E2F1 | 1.61162697 | 3.027556661 | 11.11973385 | 5.11E-26 | 2.40E-24 | 48.23594192 |
| GPX3 | -2.443587822 | 4.083610985 | -11.10110003 | 6.05E-26 | 2.83E-24 | 48.06795121 |
| GALNT2 | 1.136259749 | 4.367041561 | 11.06867274 | 8.13E-26 | 3.73E-24 | 47.77602954 |
| DSCC1 | 1.066870632 | 2.10026651 | 10.99573875 | 1.58E-25 | 7.07E-24 | 47.1214212 |
| CHRDL1 | -1.537049728 | 0.725186371 | -10.98107612 | 1.80E-25 | 8.01E-24 | 46.99014945 |
| ARTN | 1.788673912 | 2.085973205 | 10.96891565 | 2.01E-25 | 8.86E-24 | 46.88136343 |
| COL3A1 | 3.190995313 | 7.613133138 | 10.9597125 | 2.19E-25 | 9.59E-24 | 46.79908409 |
| AOX1 | -1.030657654 | 0.722278894 | -10.94083383 | 2.59E-25 | 1.13E-23 | 46.63043976 |
| AQP5 | -2.673645087 | 1.166966079 | -10.92524295 | 2.98E-25 | 1.30E-23 | 46.49130496 |
| CCNE1 | 1.150069184 | 2.269834611 | 10.90479992 | 3.59E-25 | 1.55E-23 | 46.30906074 |
| GINS2 | 1.319804556 | 2.733440906 | 10.87529999 | 4.68E-25 | 2.01E-23 | 46.04646097 |
| CHTF18 | 1.129499314 | 2.221608522 | 10.85391211 | 5.68E-25 | 2.41E-23 | 45.85635716 |
| AGTRAP | 1.263091597 | 4.866075614 | 10.79993654 | 9.22E-25 | 3.84E-23 | 45.37766983 |
| GMNN | 1.17702745 | 3.188661349 | 10.77081388 | 1.20E-24 | 4.96E-23 | 45.12003131 |
| FYCO1 | -1.180537407 | 2.500616814 | -10.74792697 | 1.47E-24 | 6.02E-23 | 44.91787364 |
| FRZB | -1.42793982 | 1.030504251 | -10.74071795 | 1.57E-24 | 6.36E-23 | 44.85425478 |
| FGD6 | 1.070198629 | 2.238951844 | 10.73961307 | 1.58E-24 | 6.38E-23 | 44.84450678 |
| C6orf58 | -2.348464439 | 0.525760297 | -10.72807982 | 1.75E-24 | 7.04E-23 | 44.74279123 |
| CENPF | 1.376112361 | 2.902387455 | 10.69657289 | 2.32E-24 | 9.10E-23 | 44.46528265 |
| CLCA4 | -3.08515238 | 2.225526537 | -10.67135355 | 2.91E-24 | 1.13E-22 | 44.2435365 |
| ALDH9A1 | -1.010780333 | 4.632810273 | -10.63724668 | 3.93E-24 | 1.51E-22 | 43.9441875 |
| FST | 2.294521526 | 4.468456386 | 10.63148956 | 4.14E-24 | 1.58E-22 | 43.89372012 |
| FKBP9 | 1.204828439 | 4.673035489 | 10.5956043 | 5.69E-24 | 2.16E-22 | 43.57955003 |
| CKMT2 | -2.009405423 | 0.883763566 | -10.59227776 | 5.86E-24 | 2.20E-22 | 43.55046187 |
| GSDME | 1.382056152 | 2.161950662 | 10.59204218 | 5.87E-24 | 2.20E-22 | 43.54840211 |
| FNDC3B | 1.218268082 | 3.002269337 | 10.59169212 | 5.89E-24 | 2.20E-22 | 43.54534146 |
| AMOT | -1.502158652 | 0.981171461 | -10.57798169 | 6.65E-24 | 2.47E-22 | 43.42552144 |
| CYP2J2 | -1.103762798 | 1.361735725 | -10.55900996 | 7.87E-24 | 2.91E-22 | 43.25988919 |
| COX7A1 | -2.093109129 | 2.50346563 | -10.54416121 | 8.97E-24 | 3.29E-22 | 43.1303889 |
| ARHGAP11A | 1.093166824 | 2.40557805 | 10.53796586 | 9.47E-24 | 3.46E-22 | 43.07639284 |
| ATP13A4 | -1.616728024 | 1.008006109 | -10.52301499 | 1.08E-23 | 3.90E-22 | 42.94617349 |
| GMDS | -1.022616068 | 2.579830552 | -10.49140435 | 1.43E-23 | 5.12E-22 | 42.67125226 |
| BOC | -1.049164358 | 1.154633134 | -10.47331481 | 1.67E-23 | 5.98E-22 | 42.51417132 |
| ABRA | -1.014253275 | 0.247522132 | -10.42151376 | 2.64E-23 | 9.32E-22 | 42.06534995 |
| CHST2 | 2.072740705 | 3.186037006 | 10.41864633 | 2.71E-23 | 9.53E-22 | 42.04054872 |
| CKAP2 | 1.064111403 | 3.528883173 | 10.41054124 | 2.90E-23 | 1.02E-21 | 41.97047012 |
| ALG1L | 1.834918762 | 3.393687659 | 10.40471405 | 3.06E-23 | 1.06E-21 | 41.92010913 |
| CEACAM7 | -2.27528547 | 1.224010098 | -10.35989817 | 4.52E-23 | 1.55E-21 | 41.5334203 |
| COL1A2 | 2.862471013 | 6.842155322 | 10.33441002 | 5.65E-23 | 1.91E-21 | 41.31399599 |
| ANXA9 | -1.65361511 | 1.949370497 | -10.31304251 | 6.80E-23 | 2.28E-21 | 41.13032446 |
| CFD | -1.815014845 | 3.348049672 | -10.3087732 | 7.06E-23 | 2.35E-21 | 41.09365678 |
| COL6A1 | 2.240730508 | 6.290529053 | 10.29972074 | 7.64E-23 | 2.53E-21 | 41.01594185 |
| APP | 1.227643228 | 6.968841182 | 10.24498029 | 1.23E-22 | 4.01E-21 | 40.54697573 |
| GCNT3 | -1.648087197 | 1.14468713 | -10.22677504 | 1.44E-22 | 4.68E-21 | 40.39138291 |
| CLSPN | 1.011251199 | 1.551486333 | 10.19340992 | 1.92E-22 | 6.17E-21 | 40.10671015 |
| ENO2 | 1.869914333 | 2.915307777 | 10.19271703 | 1.93E-22 | 6.19E-21 | 40.10080509 |
| ACTL6A | 1.215318211 | 4.378779686 | 10.18238532 | 2.11E-22 | 6.72E-21 | 40.01278614 |
| BOP1 | 1.166822317 | 4.867642131 | 10.16741758 | 2.40E-22 | 7.62E-21 | 39.88537871 |
| ATP1B3 | 1.259583739 | 7.258654235 | 10.16685276 | 2.41E-22 | 7.64E-21 | 39.88057343 |
| GJC1 | 1.023775216 | 1.188913388 | 10.12656596 | 3.41E-22 | 1.07E-20 | 39.53829195 |
| E2F7 | 1.137235978 | 1.670994412 | 10.12603092 | 3.43E-22 | 1.07E-20 | 39.53375241 |
| CSGALNACT2 | 1.028482953 | 2.861563789 | 10.11887986 | 3.65E-22 | 1.13E-20 | 39.47309495 |
| ADAM12 | 1.858730607 | 2.132968946 | 10.09402156 | 4.51E-22 | 1.38E-20 | 39.26246624 |
| COL16A1 | 1.614957863 | 3.597975756 | 10.04567124 | 6.83E-22 | 2.07E-20 | 38.85379908 |
| GALNT18 | 1.240809867 | 3.506020487 | 10.03657414 | 7.38E-22 | 2.22E-20 | 38.77705854 |
| CASQ1 | -2.365104563 | 0.882184376 | -10.01499806 | 8.88E-22 | 2.67E-20 | 38.59523937 |
| ACP5 | 1.35214705 | 4.792955609 | 10.01189099 | 9.12E-22 | 2.73E-20 | 38.56907856 |
| BBOX1-AS1 | 1.306947329 | 2.087795182 | 9.984353121 | 1.15E-21 | 3.42E-20 | 38.33745989 |
| APOD | -2.327523906 | 2.501411791 | -9.960697528 | 1.41E-21 | 4.14E-20 | 38.13884521 |
| ADAM8 | 1.536289351 | 2.888159746 | 9.957335669 | 1.45E-21 | 4.24E-20 | 38.11064505 |
| ALDH1L1 | -1.09943096 | 0.539345671 | -9.917778023 | 2.03E-21 | 5.85E-20 | 37.77931865 |
| AMPD1 | -1.482036402 | 0.588444677 | -9.913315483 | 2.11E-21 | 6.06E-20 | 37.74199857 |
| CALU | 1.187522576 | 5.30329109 | 9.908848538 | 2.19E-21 | 6.28E-20 | 37.70465329 |
| GDPD3 | -1.758976737 | 2.070633736 | -9.904148147 | 2.28E-21 | 6.50E-20 | 37.66536889 |
| CTSV | 1.867525319 | 3.809442812 | 9.904065192 | 2.28E-21 | 6.50E-20 | 37.66467569 |
| EPHX2 | -1.335790294 | 1.963041163 | -9.895775986 | 2.45E-21 | 6.95E-20 | 37.59542894 |
| BRMS1 | 1.035553747 | 5.092862601 | 9.890052521 | 2.57E-21 | 7.27E-20 | 37.54763943 |
| FCER1A | -1.640671329 | 1.521434992 | -9.885882547 | 2.66E-21 | 7.49E-20 | 37.51283324 |
| DSN1 | 1.005798973 | 3.068131905 | 9.885655796 | 2.66E-21 | 7.49E-20 | 37.51094087 |
| BCAS1 | -1.145714979 | 0.686680557 | -9.869800369 | 3.05E-21 | 8.51E-20 | 37.3786929 |
| COL5A3 | 1.721103309 | 3.319272276 | 9.857584007 | 3.38E-21 | 9.41E-20 | 37.27689836 |
| ACADSB | -1.022425063 | 1.598412186 | -9.849629404 | 3.61E-21 | 1.00E-19 | 37.2106626 |
| CDCA2 | 1.059921562 | 2.082686971 | 9.838088561 | 3.98E-21 | 1.10E-19 | 37.11463131 |
| FOXD1 | 1.227189899 | 1.773853602 | 9.79012218 | 5.96E-21 | 1.62E-19 | 36.71634372 |
| COL6A3 | 2.374924517 | 4.381419972 | 9.787605921 | 6.09E-21 | 1.65E-19 | 36.69548752 |
| CSRNP1 | -1.15797868 | 3.427824549 | -9.756390963 | 7.92E-21 | 2.11E-19 | 36.43707152 |
| ACKR1 | -1.973985031 | 2.04158408 | -9.724128925 | 1.04E-20 | 2.72E-19 | 36.17059462 |
| C7 | -1.208313413 | 0.477667214 | -9.722126056 | 1.05E-20 | 2.76E-19 | 36.15407179 |
| CA6 | -1.058764857 | 0.186158446 | -9.709065598 | 1.18E-20 | 3.07E-19 | 36.0463871 |
| AIF1L | -1.539956441 | 2.397987625 | -9.699923546 | 1.27E-20 | 3.27E-19 | 35.97107052 |
| COTL1 | 1.188480402 | 4.16302184 | 9.686398239 | 1.42E-20 | 3.64E-19 | 35.8597341 |
| DTYMK | 1.01585226 | 3.758535345 | 9.664386272 | 1.71E-20 | 4.31E-19 | 35.67877176 |
| CAVIN2 | -1.422635435 | 1.567048264 | -9.632293854 | 2.23E-20 | 5.50E-19 | 35.41545732 |
| CCN4 | 1.556272337 | 1.790412643 | 9.607264331 | 2.75E-20 | 6.70E-19 | 35.21052263 |
| ATP2A1 | -2.540832305 | 1.182253291 | -9.606273798 | 2.77E-20 | 6.74E-19 | 35.20242018 |
| FOXF2 | 1.174656442 | 1.991678166 | 9.597523951 | 2.98E-20 | 7.18E-19 | 35.13087312 |
| GPR176 | 1.536502661 | 2.168023241 | 9.591664108 | 3.13E-20 | 7.50E-19 | 35.08298329 |
| FXYD5 | 1.502441835 | 5.203461515 | 9.520424933 | 5.63E-20 | 1.33E-18 | 34.50243919 |
| CTSC | 1.347559172 | 4.622670009 | 9.520405081 | 5.63E-20 | 1.33E-18 | 34.50227784 |
| FANCE | 1.007857686 | 3.355903873 | 9.506207263 | 6.33E-20 | 1.48E-18 | 34.38694483 |
| ASPM | 1.050955658 | 2.063231267 | 9.480450143 | 7.83E-20 | 1.82E-18 | 34.17802569 |
| CLU | -2.451966969 | 3.906943871 | -9.452282121 | 9.87E-20 | 2.28E-18 | 33.95001513 |
| ANKRD35 | -1.645320537 | 1.943676607 | -9.449617182 | 1.01E-19 | 2.33E-18 | 33.92846849 |
| CDH3 | 1.688755737 | 6.117512123 | 9.445025521 | 1.05E-19 | 2.41E-18 | 33.89135407 |
| DNMT1 | 1.04739565 | 3.552432652 | 9.428556641 | 1.20E-19 | 2.74E-18 | 33.75834231 |
| CAVIN3 | 1.467376674 | 3.602236728 | 9.403844473 | 1.47E-19 | 3.33E-18 | 33.5590656 |
| CHN1 | 1.002694541 | 1.596262268 | 9.400303203 | 1.51E-19 | 3.41E-18 | 33.53053988 |
| ENPP4 | -1.07504739 | 0.870177001 | -9.400164778 | 1.51E-19 | 3.41E-18 | 33.52942499 |
| CAPN6 | -1.241498189 | 0.518745073 | -9.388337363 | 1.67E-19 | 3.74E-18 | 33.43420948 |
| FADS3 | 1.292514934 | 2.094604908 | 9.345779272 | 2.36E-19 | 5.19E-18 | 33.09231344 |
| GGT6 | -1.806321243 | 2.864917227 | -9.341803422 | 2.43E-19 | 5.35E-18 | 33.06043011 |
| CASC9 | 2.026660326 | 2.154241843 | 9.340777479 | 2.45E-19 | 5.37E-18 | 33.05220441 |
| CHST15 | 1.109919328 | 2.97930492 | 9.317503809 | 2.97E-19 | 6.43E-18 | 32.86577831 |
| COL6A2 | 2.047290402 | 6.533916836 | 9.315264721 | 3.02E-19 | 6.54E-18 | 32.84786055 |
| ATP1A2 | -1.866595926 | 0.854827699 | -9.26389554 | 4.58E-19 | 9.81E-18 | 32.43764733 |
| FSTL3 | 1.870964066 | 3.604742274 | 9.245267399 | 5.32E-19 | 1.13E-17 | 32.28929705 |
| CRACDL | -1.150865788 | 1.42101514 | -9.244739377 | 5.35E-19 | 1.14E-17 | 32.28509516 |
| FN1 | 2.806690496 | 5.502568684 | 9.187989958 | 8.45E-19 | 1.77E-17 | 31.83451346 |
| GAS7 | -1.165077775 | 1.711341212 | -9.182371132 | 8.84E-19 | 1.84E-17 | 31.79001084 |
| FLNA | 1.231089938 | 7.01482728 | 9.175751499 | 9.32E-19 | 1.93E-17 | 31.73760704 |
| ENAH | 1.064791877 | 3.606696353 | 9.142311152 | 1.22E-18 | 2.50E-17 | 31.47330126 |
| H4C9 | 1.308251439 | 2.855161563 | 9.13822374 | 1.26E-18 | 2.58E-17 | 31.44104357 |
| CRTAC1 | -1.168998327 | 0.711453258 | -9.125035824 | 1.40E-18 | 2.86E-17 | 31.3370371 |
| CXCL13 | 2.23808466 | 3.289216815 | 9.114921143 | 1.52E-18 | 3.07E-17 | 31.25734225 |
| CHPF | 1.183205791 | 5.366584139 | 9.072381004 | 2.13E-18 | 4.26E-17 | 30.92287326 |
| CYP4F12 | -1.099117841 | 0.708892493 | -9.046891471 | 2.61E-18 | 5.17E-17 | 30.72301467 |
| FITM1 | -1.382246833 | 0.750359516 | -9.020239907 | 3.23E-18 | 6.33E-17 | 30.51448788 |
| FZD6 | 1.173834855 | 4.603408457 | 8.990474499 | 4.09E-18 | 7.92E-17 | 30.28213463 |
| DTX3L | 1.071055658 | 4.388248849 | 8.988323468 | 4.16E-18 | 8.03E-17 | 30.26536535 |
| FMNL2 | 1.019080453 | 3.207685929 | 8.957832621 | 5.29E-18 | 1.01E-16 | 30.02798033 |
| GPX8 | 1.101068123 | 2.631828221 | 8.955242556 | 5.40E-18 | 1.03E-16 | 30.00784303 |
| CNTFR | -1.133747456 | 0.677085736 | -8.95479302 | 5.42E-18 | 1.03E-16 | 30.00434841 |
| BNC1 | 1.607719157 | 4.263654506 | 8.926532663 | 6.77E-18 | 1.29E-16 | 29.78491801 |
| ACP3 | -1.312155237 | 1.694810935 | -8.907606711 | 7.86E-18 | 1.48E-16 | 29.63825353 |
| COQ8A | -1.364710575 | 2.226791882 | -8.904894707 | 8.03E-18 | 1.51E-16 | 29.61725613 |
| H2BC12 | 1.510658385 | 5.840145931 | 8.773848636 | 2.24E-17 | 4.05E-16 | 28.60833498 |
| CYP27B1 | 1.266925945 | 1.707739063 | 8.765504729 | 2.39E-17 | 4.31E-16 | 28.54447462 |
| APLN | 1.241338512 | 1.868183705 | 8.741141735 | 2.89E-17 | 5.19E-16 | 28.35827283 |
| DUSP14 | 1.065964739 | 4.486743869 | 8.738347746 | 2.95E-17 | 5.29E-16 | 28.3369438 |
| FADS1 | 1.291300966 | 2.149898991 | 8.738241276 | 2.95E-17 | 5.29E-16 | 28.33613112 |
| BEX3 | 1.060423778 | 6.014038212 | 8.737783282 | 2.96E-17 | 5.30E-16 | 28.33263536 |
| CENPM | 1.167711937 | 2.904921469 | 8.722021016 | 3.35E-17 | 5.96E-16 | 28.21240973 |
| EPSTI1 | 1.621213489 | 2.969028235 | 8.716806575 | 3.49E-17 | 6.18E-16 | 28.17267289 |
| COL10A1 | 2.144567978 | 2.188784938 | 8.715131418 | 3.53E-17 | 6.24E-16 | 28.15991109 |
| H1-10 | 1.003764001 | 6.274084456 | 8.674895262 | 4.82E-17 | 8.38E-16 | 27.85393698 |
| EPHB2 | 1.108271829 | 1.573870627 | 8.674525037 | 4.83E-17 | 8.39E-16 | 27.85112658 |
| GPR153 | 1.270253753 | 3.649393487 | 8.646063111 | 6.02E-17 | 1.03E-15 | 27.63534186 |
| ESM1 | 1.060395235 | 1.464741261 | 8.636273142 | 6.49E-17 | 1.11E-15 | 27.56124298 |
| FKBP10 | 1.853939901 | 3.813407093 | 8.614745677 | 7.66E-17 | 1.29E-15 | 27.39852831 |
| FBLIM1 | 1.203810633 | 4.589143419 | 8.611638303 | 7.84E-17 | 1.32E-15 | 27.37506674 |
| FAP | 1.380902971 | 1.752540725 | 8.594975484 | 8.91E-17 | 1.49E-15 | 27.24936719 |
| GRIN2D | 1.364613746 | 1.464323622 | 8.588162865 | 9.39E-17 | 1.56E-15 | 27.19802796 |
| BPIFA2 | -1.205172553 | 0.150342379 | -8.58090361 | 9.92E-17 | 1.64E-15 | 27.14335696 |
| BGN | 1.895800461 | 6.949358702 | 8.578773971 | 1.01E-16 | 1.67E-15 | 27.12732485 |
| DUSP13 | -1.309152348 | 0.73220492 | -8.575872203 | 1.03E-16 | 1.70E-15 | 27.10548496 |
| FERMT1 | 1.379116934 | 4.408155777 | 8.574467782 | 1.04E-16 | 1.72E-15 | 27.09491673 |
| EHD2 | 1.086490998 | 5.631948901 | 8.53527185 | 1.41E-16 | 2.28E-15 | 26.80049977 |
| APOL1 | 1.927867182 | 6.124102984 | 8.529327827 | 1.47E-16 | 2.37E-15 | 26.75594146 |
| AADAC | -1.409036268 | 0.772097077 | -8.526687491 | 1.50E-16 | 2.41E-15 | 26.73615625 |
| FJX1 | 1.149142325 | 3.108380396 | 8.515978993 | 1.63E-16 | 2.61E-15 | 26.65596058 |
| CD109 | 1.141146152 | 3.683095159 | 8.509483169 | 1.71E-16 | 2.73E-15 | 26.60735099 |
| CTSZ | 1.157966783 | 6.204029895 | 8.50102584 | 1.83E-16 | 2.90E-15 | 26.54410548 |
| GALNT12 | -1.188907045 | 1.707978562 | -8.497078756 | 1.88E-16 | 2.98E-15 | 26.51460489 |
| BICDL2 | -1.415031008 | 2.467391238 | -8.496830007 | 1.88E-16 | 2.98E-15 | 26.51274608 |
| ACER1 | -1.252223322 | 0.767755187 | -8.482831187 | 2.10E-16 | 3.29E-15 | 26.40820532 |
| H2BC5 | 1.311918803 | 3.849764182 | 8.473456896 | 2.25E-16 | 3.52E-15 | 26.33827342 |
| CLDN10 | -1.523502002 | 0.720352894 | -8.463579426 | 2.43E-16 | 3.78E-15 | 26.26465184 |
| C12orf75 | 1.389976077 | 3.692364001 | 8.453909537 | 2.61E-16 | 4.06E-15 | 26.19264115 |
| BEX4 | -1.521232374 | 2.870409973 | -8.450811994 | 2.67E-16 | 4.15E-15 | 26.1695874 |
| ENO3 | -2.15497949 | 1.596286121 | -8.426692763 | 3.21E-16 | 4.93E-15 | 25.99029933 |
| ADAMTS12 | 1.283694087 | 1.470598486 | 8.424225422 | 3.27E-16 | 5.01E-15 | 25.97198074 |
| H3C10 | 1.219621327 | 1.858402255 | 8.42234539 | 3.31E-16 | 5.07E-15 | 25.95802535 |
| DCBLD2 | 1.155555768 | 2.537436221 | 8.419109374 | 3.40E-16 | 5.19E-15 | 25.93401015 |
| APOBEC3B | 1.566083503 | 3.193722315 | 8.396571502 | 4.02E-16 | 6.08E-15 | 25.76694819 |
| FAM89A | 1.169455628 | 3.440316328 | 8.386475908 | 4.34E-16 | 6.53E-15 | 25.69222625 |
| CMYA5 | -1.880926915 | 1.117688511 | -8.364330581 | 5.13E-16 | 7.68E-15 | 25.52856129 |
| FNDC4 | -1.008421219 | 1.706210262 | -8.326395103 | 6.82E-16 | 1.01E-14 | 25.24897459 |
| ADPRHL1 | -1.106247512 | 1.278313199 | -8.310824471 | 7.66E-16 | 1.13E-14 | 25.13450216 |
| CCL28 | -1.205806733 | 1.274383708 | -8.283251589 | 9.41E-16 | 1.36E-14 | 24.93219798 |
| EYA2 | -1.922083377 | 2.001673152 | -8.283173086 | 9.41E-16 | 1.36E-14 | 24.93162274 |
| AEBP1 | 1.882031164 | 5.337812149 | 8.281335648 | 9.54E-16 | 1.38E-14 | 24.91815991 |
| AJUBA | 1.091042763 | 3.741030803 | 8.278080803 | 9.78E-16 | 1.41E-14 | 24.8943175 |
| C6orf141 | 1.221004806 | 1.696218435 | 8.267537805 | 1.06E-15 | 1.52E-14 | 24.81713771 |
| CLDN8 | -1.37143127 | 0.763329998 | -8.264735559 | 1.08E-15 | 1.54E-14 | 24.79663676 |
| FEZ1 | 1.396359136 | 2.372350778 | 8.254339021 | 1.17E-15 | 1.66E-14 | 24.72062379 |
| AMIGO2 | 1.541736891 | 2.732300402 | 8.251565549 | 1.19E-15 | 1.69E-14 | 24.70035844 |
| GATM | -1.182036429 | 1.477982311 | -8.225972056 | 1.44E-15 | 2.03E-14 | 24.51360012 |
| GABRP | -2.336146701 | 1.608652235 | -8.212547703 | 1.59E-15 | 2.22E-14 | 24.4158215 |
| CH25H | -1.295618596 | 1.766826408 | -8.165879251 | 2.25E-15 | 3.07E-14 | 24.07687126 |
| CST4 | -1.315659841 | 0.40278837 | -8.146654597 | 2.59E-15 | 3.51E-14 | 23.9376818 |
| EHF | -1.759170192 | 4.149807441 | -8.139399315 | 2.73E-15 | 3.68E-14 | 23.88521902 |
| ADGRF1 | -1.140946019 | 0.862904052 | -8.137097077 | 2.78E-15 | 3.73E-14 | 23.86857922 |
| FUT3 | -1.683561731 | 2.661589067 | -8.13362685 | 2.85E-15 | 3.82E-14 | 23.84350455 |
| CENPW | 1.181365759 | 4.725551355 | 8.125400896 | 3.03E-15 | 4.05E-14 | 23.78410001 |
| FADD | 1.374525163 | 3.497144933 | 8.116979357 | 3.22E-15 | 4.30E-14 | 23.72333172 |
| EMP3 | 1.230114315 | 4.448649802 | 8.112302128 | 3.33E-15 | 4.44E-14 | 23.68960299 |
| DUSP9 | 1.31241708 | 1.543540936 | 8.040292297 | 5.65E-15 | 7.28E-14 | 23.1722452 |
| AFAP1L2 | 1.144664868 | 3.452986594 | 7.975214667 | 9.06E-15 | 1.15E-13 | 22.70781342 |
| C5orf66-AS1 | -1.625556288 | 1.553026168 | -7.953105054 | 1.06E-14 | 1.34E-13 | 22.55070393 |
| DMBT1 | -2.081217582 | 0.877154885 | -7.901969827 | 1.54E-14 | 1.90E-13 | 22.18866201 |
| DSG1-AS1 | -1.013167924 | 0.982041906 | -7.898014408 | 1.58E-14 | 1.95E-13 | 22.16073436 |
| GREM1 | 1.366994415 | 1.926857443 | 7.892062134 | 1.65E-14 | 2.03E-13 | 22.1187286 |
| ABLIM1 | -1.155044514 | 4.534535829 | -7.858287332 | 2.10E-14 | 2.56E-13 | 21.88085262 |
| BSPRY | -1.423932533 | 2.629596348 | -7.85380719 | 2.17E-14 | 2.64E-13 | 21.84935984 |
| COL4A6 | 1.430233199 | 1.910428344 | 7.85288673 | 2.19E-14 | 2.65E-13 | 21.84289132 |
| F2R | 1.189956979 | 3.344912133 | 7.829316265 | 2.59E-14 | 3.11E-13 | 21.67745545 |
| FOLR1 | -1.428445368 | 0.74819299 | -7.810011031 | 2.97E-14 | 3.55E-13 | 21.54225122 |
| H2BC9 | 1.087585555 | 2.009306027 | 7.799734856 | 3.20E-14 | 3.81E-13 | 21.47039049 |
| CHST7 | 1.069215945 | 1.897817159 | 7.773214546 | 3.86E-14 | 4.58E-13 | 21.28528419 |
| AZGP1 | -2.26910186 | 1.669065447 | -7.766816589 | 4.04E-14 | 4.78E-13 | 21.24070314 |
| DEGS2 | -1.190073251 | 1.352976463 | -7.748995327 | 4.59E-14 | 5.42E-13 | 21.11667901 |
| CBX2 | 1.079501827 | 2.111700128 | 7.739424012 | 4.91E-14 | 5.78E-13 | 21.05016306 |
| CYP27C1 | 1.066633866 | 1.565591467 | 7.718636153 | 5.69E-14 | 6.66E-13 | 20.90592432 |
| ANXA1 | -1.597149034 | 8.126173989 | -7.715100539 | 5.83E-14 | 6.80E-13 | 20.88142301 |
| CACNA1S | -1.272889814 | 0.588603494 | -7.708199779 | 6.12E-14 | 7.10E-13 | 20.83362762 |
| DDX60 | 1.426910213 | 3.241099235 | 7.701168129 | 6.43E-14 | 7.44E-13 | 20.78496095 |
| CEACAM6 | -2.290318152 | 4.126572657 | -7.685785497 | 7.17E-14 | 8.24E-13 | 20.67862061 |
| EIF4EBP3 | -1.028743784 | 1.928644974 | -7.667369417 | 8.16E-14 | 9.30E-13 | 20.55153453 |
| CPXM1 | 1.498559373 | 2.777230144 | 7.654770676 | 8.92E-14 | 1.01E-12 | 20.464734 |
| COX6A2 | -2.521477247 | 1.572961598 | -7.649328413 | 9.26E-14 | 1.05E-12 | 20.42727438 |
| FTL | 1.095501183 | 10.60027032 | 7.637942666 | 1.00E-13 | 1.13E-12 | 20.3489745 |
| C4orf48 | 1.126604244 | 2.059866083 | 7.634250144 | 1.03E-13 | 1.16E-12 | 20.32360116 |
| CGN | -1.095481464 | 1.646564944 | -7.629143786 | 1.07E-13 | 1.20E-12 | 20.28852885 |
| CXCL17 | -2.419773923 | 3.69499761 | -7.628351805 | 1.07E-13 | 1.20E-12 | 20.28309093 |
| CLEC11A | 1.227695017 | 2.796159742 | 7.60601636 | 1.25E-13 | 1.40E-12 | 20.12991808 |
| DNAH17 | 1.258079078 | 1.442684833 | 7.579556983 | 1.51E-13 | 1.66E-12 | 19.94893278 |
| BLNK | -1.076223703 | 2.227052311 | -7.56492896 | 1.67E-13 | 1.84E-12 | 19.84909399 |
| ALOX12 | -1.577041934 | 1.805295214 | -7.547016491 | 1.89E-13 | 2.07E-12 | 19.72705065 |
| C10orf71 | -1.15532905 | 0.51594737 | -7.505167982 | 2.53E-13 | 2.74E-12 | 19.44283651 |
| EN1 | 1.201258649 | 1.37170819 | 7.503436255 | 2.56E-13 | 2.77E-12 | 19.43110308 |
| CRYAB | -2.04640101 | 4.33691744 | -7.50280912 | 2.57E-13 | 2.77E-12 | 19.42685443 |
| CAV1 | 1.644429749 | 5.886537407 | 7.499078229 | 2.64E-13 | 2.84E-12 | 19.40158473 |
| F2RL2 | 1.238093831 | 1.85647072 | 7.36014964 | 6.84E-13 | 7.08E-12 | 18.46789309 |
| CTSL | 1.190508956 | 5.17736518 | 7.352501073 | 7.21E-13 | 7.44E-12 | 18.4169035 |
| DDX58 | 1.242344497 | 3.387983157 | 7.350160769 | 7.33E-13 | 7.55E-12 | 18.40131039 |
| CD24 | -1.571953853 | 6.656902472 | -7.330635368 | 8.37E-13 | 8.52E-12 | 18.27137383 |
| FOXP3 | 1.001839138 | 1.801234847 | 7.329390863 | 8.44E-13 | 8.58E-12 | 18.26310156 |
| FUT2 | -1.185736774 | 2.978828634 | -7.325299448 | 8.67E-13 | 8.81E-12 | 18.23591388 |
| GAST | 1.563105055 | 1.59200994 | 7.319261038 | 9.04E-13 | 9.15E-12 | 18.19581103 |
| ALDH1A1 | -2.441265922 | 3.007232538 | -7.316656479 | 9.20E-13 | 9.28E-12 | 18.17852176 |
| FHL1 | -1.984111209 | 2.960990316 | -7.308547262 | 9.72E-13 | 9.80E-12 | 18.12472441 |
| FCGR2A | 1.038154144 | 2.398080621 | 7.297512014 | 1.05E-12 | 1.05E-11 | 18.05159407 |
| GALNT6 | 1.415615961 | 3.133614154 | 7.290838569 | 1.10E-12 | 1.10E-11 | 18.00741329 |
| DDIT4L | -1.371444929 | 0.962128655 | -7.290385551 | 1.10E-12 | 1.10E-11 | 18.00441534 |
| EPS8L1 | -1.331226846 | 3.220370863 | -7.236991627 | 1.57E-12 | 1.54E-11 | 17.65214104 |
| EGR1 | -1.405317704 | 6.243895513 | -7.230885891 | 1.64E-12 | 1.60E-11 | 17.61199326 |
| BASP1 | 1.465263352 | 4.671325668 | 7.216750877 | 1.80E-12 | 1.75E-11 | 17.5191567 |
| CCL11 | 1.207644521 | 1.370140195 | 7.203771629 | 1.97E-12 | 1.90E-11 | 17.43404286 |
| DNASE1L3 | -1.16712135 | 1.423124422 | -7.196306081 | 2.07E-12 | 1.99E-11 | 17.38514338 |
| FOS | -1.635463087 | 6.56636273 | -7.160909176 | 2.62E-12 | 2.49E-11 | 17.15386266 |
| BPIFB2 | -2.467593539 | 1.00367965 | -7.153697541 | 2.75E-12 | 2.60E-11 | 17.10685792 |
| DSG2 | 1.273460698 | 4.471380652 | 7.147929932 | 2.86E-12 | 2.69E-11 | 17.06929338 |
| CLDN17 | -1.450522454 | 1.025894218 | -7.13253207 | 3.17E-12 | 2.97E-11 | 16.96912962 |
| APOBEC2 | -1.766114029 | 1.130821181 | -7.098090872 | 3.98E-12 | 3.69E-11 | 16.74573576 |
| CALD1 | 1.060211829 | 4.206062989 | 7.09702372 | 4.01E-12 | 3.71E-11 | 16.73882827 |
| DUSP26 | -1.358102137 | 0.860722027 | -7.082588971 | 4.41E-12 | 4.06E-11 | 16.64547931 |
| FCGR3A | 1.462496344 | 3.215199011 | 7.054927717 | 5.29E-12 | 4.83E-11 | 16.46703602 |
| GBP5 | 1.796294438 | 2.545570766 | 7.04926015 | 5.49E-12 | 5.00E-11 | 16.43054606 |
| ASPN | 1.775599324 | 2.986588426 | 7.041095929 | 5.79E-12 | 5.24E-11 | 16.37802454 |
| ACTN1 | 1.03878682 | 5.249482804 | 7.014273128 | 6.91E-12 | 6.19E-11 | 16.20582647 |
| CLEC7A | 1.088990302 | 2.286392656 | 7.013971789 | 6.92E-12 | 6.19E-11 | 16.20389503 |
| COL18A1 | 1.005309337 | 4.493829105 | 7.006438871 | 7.27E-12 | 6.48E-11 | 16.15563508 |
| GNLY | 1.439592192 | 2.352024551 | 6.99646333 | 7.76E-12 | 6.88E-11 | 16.09179287 |
| CDH11 | 1.213826901 | 1.908126352 | 6.9856992 | 8.33E-12 | 7.31E-11 | 16.02298886 |
| CDK6 | 1.030930988 | 3.041458111 | 6.968701331 | 9.31E-12 | 8.10E-11 | 15.91451897 |
| CYP2C18 | -1.379717481 | 1.796332841 | -6.95252908 | 1.03E-11 | 8.93E-11 | 15.81152251 |
| DUSP1 | -1.334028821 | 6.868852854 | -6.948761005 | 1.06E-11 | 9.15E-11 | 15.78755343 |
| EMILIN1 | 1.441416567 | 3.259610909 | 6.943544429 | 1.10E-11 | 9.42E-11 | 15.75438822 |
| ANO1 | 1.794645251 | 4.21886988 | 6.882177635 | 1.63E-11 | 1.39E-10 | 15.36580471 |
| CYSRT1 | -1.969250097 | 3.740148514 | -6.877717763 | 1.68E-11 | 1.42E-10 | 15.33767682 |
| CHL1 | -1.097759171 | 1.057905512 | -6.870172195 | 1.76E-11 | 1.49E-10 | 15.29012266 |
| CKM | -3.195979289 | 2.713836858 | -6.830688241 | 2.27E-11 | 1.89E-10 | 15.04199932 |
| CMPK2 | 1.183640556 | 2.242373578 | 6.789456371 | 2.96E-11 | 2.43E-10 | 14.78417599 |
| ADAM19 | 1.091023829 | 2.257824339 | 6.789263491 | 2.96E-11 | 2.43E-10 | 14.782973 |
| CCL2 | -1.306867152 | 3.186599658 | -6.777280994 | 3.20E-11 | 2.61E-10 | 14.70829472 |
| CLDN4 | -1.55187418 | 4.876037837 | -6.739469143 | 4.07E-11 | 3.29E-10 | 14.47337048 |
| ARSI | 1.405484019 | 2.837482765 | 6.734867989 | 4.19E-11 | 3.38E-10 | 14.44485935 |
| GALNT5 | -1.118051293 | 1.322202359 | -6.73210576 | 4.26E-11 | 3.43E-10 | 14.42775106 |
| CRAT | -1.260578691 | 2.88135231 | -6.72453141 | 4.47E-11 | 3.59E-10 | 14.38086858 |
| COL11A1 | 1.669156575 | 1.809932582 | 6.70963668 | 4.92E-11 | 3.92E-10 | 14.28880578 |
| CEACAM5 | -2.343075286 | 3.376891403 | -6.688858443 | 5.61E-11 | 4.45E-10 | 14.16066622 |
| GJB3 | 1.059616644 | 5.592492353 | 6.688646446 | 5.62E-11 | 4.45E-10 | 14.15936057 |
| H2AC8 | 1.09506383 | 2.298339388 | 6.66046166 | 6.71E-11 | 5.26E-10 | 13.98608759 |
| BNIPL | -1.36176167 | 2.971281403 | -6.6601823 | 6.72E-11 | 5.26E-10 | 13.98437326 |
| GPR68 | 1.147299238 | 3.269041241 | 6.653336798 | 7.01E-11 | 5.47E-10 | 13.94238397 |
| ANKRD2 | -1.136718434 | 0.995058952 | -6.648079325 | 7.25E-11 | 5.64E-10 | 13.91016029 |
| CYCSP6 | 1.280359012 | 1.262665437 | 6.642736819 | 7.50E-11 | 5.82E-10 | 13.87743758 |
| CSPG4 | 1.552136251 | 3.449748105 | 6.633184027 | 7.96E-11 | 6.17E-10 | 13.81898264 |
| APOC1 | 1.696352912 | 3.397335891 | 6.605346554 | 9.47E-11 | 7.25E-10 | 13.64904871 |
| CXCL10 | 2.458976696 | 4.9361939 | 6.590950402 | 1.04E-10 | 7.88E-10 | 13.56140578 |
| FABP3 | -1.430777515 | 2.28955504 | -6.532627014 | 1.49E-10 | 1.11E-09 | 13.20800358 |
| GJA1 | 1.387724077 | 6.849387807 | 6.522851152 | 1.58E-10 | 1.18E-09 | 13.14903035 |
| CSF2 | 1.360046742 | 1.444554065 | 6.492911809 | 1.90E-10 | 1.40E-09 | 12.96888962 |
| DUSP5 | -1.004709163 | 4.23199794 | -6.487354215 | 1.97E-10 | 1.44E-09 | 12.93552833 |
| CYP4X1 | -1.363867877 | 1.561052528 | -6.482166869 | 2.03E-10 | 1.48E-09 | 12.90441164 |
| C1orf116 | -1.102949453 | 4.089747553 | -6.476684292 | 2.10E-10 | 1.53E-09 | 12.87154712 |
| APOE | 1.681119871 | 5.153312627 | 6.461583899 | 2.31E-10 | 1.67E-09 | 12.78115307 |
| DIO2 | -1.199325672 | 2.611797083 | -6.425754583 | 2.87E-10 | 2.06E-09 | 12.56739494 |
| DEFB1 | -1.9750225 | 4.760911496 | -6.425339357 | 2.88E-10 | 2.06E-09 | 12.56492368 |
| COL8A1 | 1.148825288 | 1.755967367 | 6.412762133 | 3.11E-10 | 2.22E-09 | 12.49013371 |
| ACTN2 | -2.143707104 | 1.525308857 | -6.410219681 | 3.16E-10 | 2.26E-09 | 12.47503041 |
| BPIFB1 | -2.564473616 | 1.413878436 | -6.38645022 | 3.65E-10 | 2.58E-09 | 12.334078 |
| FBN2 | 1.233028191 | 1.381071256 | 6.378412951 | 3.83E-10 | 2.71E-09 | 12.28651897 |
| DLX5 | 1.105714222 | 2.48929617 | 6.36271494 | 4.22E-10 | 2.95E-09 | 12.19377749 |
| ACTA1 | -3.370314766 | 3.081428121 | -6.345054916 | 4.69E-10 | 3.26E-09 | 12.08967982 |
| CHI3L2 | -1.228240906 | 1.609293938 | -6.336196174 | 4.95E-10 | 3.43E-09 | 12.03755548 |
| DDIT4 | 1.208175561 | 6.112546706 | 6.29546065 | 6.33E-10 | 4.33E-09 | 11.79867819 |
| FCER1G | 1.133102934 | 4.234623959 | 6.293612844 | 6.40E-10 | 4.37E-09 | 11.78787399 |
| CNGB1 | 1.106420641 | 1.191810292 | 6.292024503 | 6.46E-10 | 4.40E-09 | 11.77858908 |
| GAMT | -1.077125371 | 2.897051415 | -6.18287066 | 1.24E-09 | 8.25E-09 | 11.14537112 |
| ADH1C | -1.315702714 | 0.783097135 | -6.107289933 | 1.93E-09 | 1.26E-08 | 10.71255227 |
| C1S | 1.03783269 | 5.533566259 | 6.091185757 | 2.12E-09 | 1.37E-08 | 10.62092921 |
| CXCL9 | 2.021675888 | 4.034919003 | 6.075064402 | 2.33E-09 | 1.50E-08 | 10.52941957 |
| BPIFA1 | -1.761476063 | 0.803054251 | -6.073306535 | 2.36E-09 | 1.51E-08 | 10.51945418 |
| CCL5 | 1.471944585 | 4.717052445 | 6.068626977 | 2.42E-09 | 1.55E-08 | 10.4929379 |
| FAT1 | 1.126509011 | 3.896991927 | 6.054288777 | 2.63E-09 | 1.68E-08 | 10.41180287 |
| EEF1A2 | -1.989611116 | 2.76335888 | -6.040564664 | 2.85E-09 | 1.81E-08 | 10.33429967 |
| GPNMB | 1.338212571 | 6.235506602 | 6.028493908 | 3.06E-09 | 1.93E-08 | 10.26626035 |
| FADS2 | 1.207795935 | 2.293444369 | 5.995147473 | 3.71E-09 | 2.32E-08 | 10.07891469 |
| CTSK | 1.316736768 | 4.951855968 | 5.955020321 | 4.67E-09 | 2.87E-08 | 9.854680081 |
| CXCL12 | -1.128455739 | 2.223160003 | -5.93571897 | 5.22E-09 | 3.19E-08 | 9.747292403 |
| DPT | -1.327715983 | 2.510721946 | -5.891957193 | 6.70E-09 | 4.02E-08 | 9.504947199 |
| FOSB | -1.518335138 | 3.581195028 | -5.83743964 | 9.13E-09 | 5.40E-08 | 9.205245331 |
| AQP3 | -1.789940655 | 7.268072372 | -5.809917468 | 1.07E-08 | 6.25E-08 | 9.054878983 |
| CXCL11 | 1.862005241 | 2.703577971 | 5.766362145 | 1.36E-08 | 7.83E-08 | 8.818198147 |
| FNDC1 | 1.201639367 | 1.840451403 | 5.757926517 | 1.43E-08 | 8.17E-08 | 8.772540635 |
| HAS3 | 1.317360689 | 4.815027332 | 5.694619684 | 2.03E-08 | 1.14E-07 | 8.43178343 |
| CXCL8 | 1.58394096 | 4.256163026 | 5.608858981 | 3.25E-08 | 1.77E-07 | 7.975498723 |
| ECHDC3 | -1.094623963 | 1.966319672 | -5.602893052 | 3.36E-08 | 1.83E-07 | 7.943986383 |
| GBP6 | -1.490270963 | 4.683377716 | -5.555779029 | 4.34E-08 | 2.32E-07 | 7.696177353 |
| CLIC3 | -1.433940099 | 4.253004771 | -5.538269698 | 4.77E-08 | 2.55E-07 | 7.604557853 |
| GBP1 | 1.209125757 | 4.391881629 | 5.456510765 | 7.39E-08 | 3.85E-07 | 7.180166678 |
| FOXA1 | -1.173867156 | 1.538396909 | -5.431863745 | 8.43E-08 | 4.33E-07 | 7.053338197 |
| ACKR3 | 1.200042423 | 4.83369004 | 5.396121952 | 1.02E-07 | 5.16E-07 | 6.870333052 |
| CYP4F22 | -1.326458201 | 1.865621409 | -5.3640316 | 1.21E-07 | 6.05E-07 | 6.706948226 |
| COL22A1 | 1.078238173 | 1.25097839 | 5.358012661 | 1.25E-07 | 6.22E-07 | 6.676400931 |
| CSTB | -1.15031637 | 8.213933681 | -5.341066563 | 1.36E-07 | 6.78E-07 | 6.590561816 |
| DKK1 | 1.261621572 | 2.202363814 | 5.211379274 | 2.67E-07 | 1.27E-06 | 5.941759712 |
| ALDH3A1 | -1.953874651 | 4.194810442 | -5.188189019 | 3.00E-07 | 1.42E-06 | 5.827261568 |
| ELF3 | -1.248098355 | 3.700705377 | -5.176047114 | 3.20E-07 | 1.50E-06 | 5.767497203 |
| CD248 | 1.020527039 | 3.826263561 | 5.173591372 | 3.24E-07 | 1.52E-06 | 5.755425066 |
| GZMB | 1.077595058 | 2.662153267 | 5.162065796 | 3.43E-07 | 1.60E-06 | 5.698836032 |
| GSTA1 | -1.788601217 | 1.636444406 | -5.159202395 | 3.48E-07 | 1.63E-06 | 5.68479485 |
| CDKN2A | 1.553384233 | 2.33281229 | 5.155374077 | 3.55E-07 | 1.66E-06 | 5.666033051 |
| EPHX3 | -1.486890088 | 2.499487761 | -5.047085518 | 6.13E-07 | 2.77E-06 | 5.140570918 |
| FCGBP | -1.048222761 | 1.866088119 | -5.030043874 | 6.68E-07 | 3.00E-06 | 5.058801247 |
| GDPD2 | 1.069972047 | 1.598630798 | 5.021512652 | 6.97E-07 | 3.12E-06 | 5.017961092 |
| AFAP1-AS1 | 1.079342711 | 1.036735539 | 4.994140235 | 7.98E-07 | 3.54E-06 | 4.887351959 |
| A2ML1 | -1.607885712 | 4.400016649 | -4.989625878 | 8.16E-07 | 3.62E-06 | 4.865873945 |
| C15orf48 | -1.015772506 | 3.607078059 | -4.972099424 | 8.90E-07 | 3.93E-06 | 4.782656013 |
| ADH7 | -1.701706851 | 2.890922335 | -4.88570919 | 1.36E-06 | 5.84E-06 | 4.37637346 |
| DES | -2.583452107 | 3.419233454 | -4.883691054 | 1.37E-06 | 5.89E-06 | 4.366960267 |
| CSRP3 | -1.795735783 | 1.782097721 | -4.727577761 | 2.90E-06 | 1.18E-05 | 3.649619879 |
| CRCT1 | -1.92064233 | 4.431945353 | -4.706831576 | 3.20E-06 | 1.29E-05 | 3.555904249 |
| ATP12A | -1.07796404 | 1.350900762 | -4.582953771 | 5.69E-06 | 2.20E-05 | 3.004234965 |
| DAPL1 | -1.353039326 | 2.75243421 | -4.577442697 | 5.84E-06 | 2.25E-05 | 2.980008237 |
| CLDN7 | -1.076012674 | 4.557467971 | -4.55096531 | 6.60E-06 | 2.52E-05 | 2.863989655 |
| COL17A1 | 1.245915757 | 6.960790166 | 4.512810864 | 7.85E-06 | 2.96E-05 | 2.697901267 |
| CCL20 | 1.284138044 | 2.933473319 | 4.484423998 | 8.93E-06 | 3.34E-05 | 2.575173046 |
| AMTN | 1.653616298 | 2.587440517 | 4.464935605 | 9.75E-06 | 3.63E-05 | 2.491332798 |
| FLNC | -1.193383733 | 2.318476035 | -4.377840927 | 1.44E-05 | 5.23E-05 | 2.120796022 |
| CSAG1 | 1.065583866 | 1.076447923 | 4.351812851 | 1.61E-05 | 5.81E-05 | 2.011380907 |
| CCNA1 | 1.063208818 | 1.703238705 | 4.324158349 | 1.82E-05 | 6.50E-05 | 1.895795323 |
| EPCAM | 1.113469264 | 3.849063146 | 4.250316905 | 2.51E-05 | 8.78E-05 | 1.590537425 |
| CCL18 | 1.119897257 | 3.576747801 | 4.210472796 | 2.98E-05 | 0.000103083 | 1.427865856 |
| CNFN | -1.660065598 | 7.05715698 | -4.172100542 | 3.51E-05 | 0.000120187 | 1.272560011 |
| CASP14 | 1.535459806 | 2.498868283 | 4.156993103 | 3.75E-05 | 0.000127582 | 1.211780665 |
| FAM83A | 1.083904692 | 4.919197659 | 3.962176939 | 8.42E-05 | 0.000272212 | 0.446589151 |
| AGR2 | -1.115125709 | 2.43546184 | -3.930610543 | 9.57E-05 | 0.000307124 | 0.325862359 |
| H19 | -1.304102341 | 4.460478354 | -3.737083066 | 0.0002059 | 0.000622305 | -0.394305539 |
| ASPRV1 | 1.210330186 | 2.521737492 | 3.565443322 | 0.000395308 | 0.00113021 | -1.004109328 |
| CES1 | -1.10610493 | 1.906332949 | -3.09227281 | 0.002088392 | 0.005208394 | -2.542882104 |
| FDCSP | -1.552542875 | 2.8844584 | -3.07780762 | 0.002190613 | 0.005439008 | -2.58660646 |
| FAM25A | -1.15798085 | 3.990304133 | -2.927420506 | 0.003561205 | 0.008465797 | -3.029440458 |
