# Supplementary material for: Identification of Prognostic Markers for Head and NeckSquamous Cell Carcinoma Based on Glycolysis-Related Genes
Source: Evid Based Complement Alternat Med. 2022 Jul 7;2022:2762595. doi: 10.1155/2022/2762595 (PMC9283050; doi:10.1155/2022/2762595)
Supplement: Supplementary Materials — Table S1: the clinical characteristics of the HNSCC samples in the training and testing sets. Table S2: a total of 505 DEGs between the HNSCC and normal samples. Table S3: 288 glycolysis-related genes. Figure S1: the correlation between the risk score and clinicopathological characteristics. [file 2762595.f1.zip › 2762595.f1/Table S3 (1).docx]

Table S3 288 glycolysis-related genes

| ABCB6 | CXCR4 | HOMER1 | PGM2 | VEGFA | GCKR |
| --- | --- | --- | --- | --- | --- |
| ADORA2B | CYB5A | HS2ST1 | PHKA2 | VLDLR | GNPDA2 |
| AGL | DCN | HS6ST2 | PKM | XYLT2 | NDC1 |
| AGRN | DDIT4 | HSPA5 | PKP2 | ZNF292 | NUP107 |
| AK3 | DEPDC1 | IDH1 | PLOD1 | ACSS1 | NUP133 |
| AK4 | DLD | IDUA | PLOD2 | ACSS2 | NUP153 |
| AKR1A1 | DPYSL4 | IER3 | PMM2 | ADH1A | NUP155 |
| ALDH7A1 | DSC2 | IGFBP3 | POLR3K | ADH1B | NUP160 |
| ALDH9A1 | ECD | IL13RA1 | PPFIA4 | ADH1C | NUP188 |
| ALDOA | EFNA3 | IRS2 | PPIA | ADH4 | NUP205 |
| ALDOB | EGFR | ISG20 | PPP2CB | ADH5 | NUP210 |
| ALG1 | EGLN3 | KDELR3 | PRPS1 | ADH6 | NUP214 |
| ANG | ELF3 | KIF20A | PSMC4 | ADH7 | NUP35 |
| ANGPTL4 | ENO1 | KIF2A | PYGB | ALDH1A3 | NUP37 |
| ANKZF1 | ENO2 | LCT | PYGL | ALDH1B1 | NUP42 |
| ARPP19 | ERO1A | LDHA | QSOX1 | ALDH2 | NUP43 |
| ARTN | EXT1 | LDHC | RARS1 | ALDH3A1 | NUP50 |
| AURKA | EXT2 | LHPP | RBCK1 | ALDH3A2 | NUP54 |
| B3GALT6 | FAM162A | LHX9 | RPE | ALDH3B1 | NUP58 |
| B3GAT1 | FBP2 | MDH1 | RRAGD | ALDH3B2 | NUP62 |
| B3GAT3 | FKBP4 | MDH2 | SAP30 | ALDOC | NUP85 |
| B3GNT3 | FUT8 | ME1 | SDC1 | BPGM | NUP88 |
| B4GALT1 | G6PD | ME2 | SDC2 | DLAT | NUP93 |
| B4GALT2 | GAL3ST1 | MED24 | SDC3 | ENO3 | NUP98 |
| B4GALT4 | GALE | MERTK | SDHC | FBP1 | PFKFB2 |
| B4GALT7 | GALK1 | MET | SLC16A3 | G6PC | PFKFB3 |
| BIK | GALK2 | MIF | SLC25A10 | G6PC2 | PFKFB4 |
| BPNT1 | GAPDHS | MIOX | SLC25A13 | GALM | PGM2L1 |
| CACNA1H | GCLC | MPI | SLC35A3 | GAPDH | PGP |
| CAPN5 | GFPT1 | MXI1 | SLC37A4 | GCK | POM121 |
| CASP6 | GLCE | NANP | SOD1 | GPI | POM121C |
| CD44 | GLRX | NASP | SOX9 | HK1 | PPP2CA |
| CDK1 | GMPPA | NDST3 | SPAG4 | HK3 | PPP2R1A |
| CENPA | GMPPB | NDUFV3 | SRD5A3 | LDHAL6A | PPP2R1B |
| CHPF | GNE | NOL3 | STC1 | LDHAL6B | PPP2R5D |
| CHPF2 | GNPDA1 | NSDHL | STC2 | LDHB | PRKACA |
| CHST1 | GOT1 | NT5E | STMN1 | PCK1 | PRKACB |
| CHST12 | GOT2 | P4HA1 | TALDO1 | PCK2 | PRKACG |
| CHST2 | GPC1 | P4HA2 | TFF3 | PDHA1 | RAE1 |
| CHST4 | GPC3 | PAM | TGFA | PDHA2 | RANBP2 |
| CHST6 | GPC4 | PAXIP1 | TGFBI | PDHB | SEC13 |
| CITED2 | GPR87 | PC | TKTL1 | PFKL | SEH1L |
| CLDN3 | GUSB | PDK3 | TPBG | PFKM | TPR |
| CLDN9 | GYS1 | PFKFB1 | TPI1 | PGAM4 |  |
| CLN6 | GYS2 | PFKP | TPST1 | PGK2 |  |
| COG2 | HAX1 | PGAM1 | TSTA3 | PGM1 |  |
| COL5A1 | HDLBP | PGAM2 | TXN | PKLR |  |
| COPB2 | HK2 | PGK1 | UGP2 | AAAS |  |
| CTH | HMMR | PGLS | VCAN | ADPGK |  |
