# Supplementary material for: Identification of Prognostic Markers for Head and NeckSquamous Cell Carcinoma Based on Glycolysis-Related Genes
Source: Evid Based Complement Alternat Med. 2022 Jul 7;2022:2762595. doi: 10.1155/2022/2762595 (PMC9283050; doi:10.1155/2022/2762595)
Supplement: Supplementary Materials — Table S1: the clinical characteristics of the HNSCC samples in the training and testing sets. Table S2: a total of 505 DEGs between the HNSCC and normal samples. Table S3: 288 glycolysis-related genes. Figure S1: the correlation between the risk score and clinicopathological characteristics. [file 2762595.f1.zip › 2762595.f1/TableS1.docx]

|  | level | TCGA_train | TCGA_test | p |
| --- | --- | --- | --- | --- |
| n |  | 350 | 150 |  |
| fustat (%) | Alive | 200 (57.1) | 83 (55.3) | 0.783 |
|  | Dead | 150 (42.9) | 67 (44.7) |  |
| age (%) | <=60 | 172 (49.1) | 72 (48.0) | 0.306 |
|  | >60 | 178 (50.9) | 77 (51.3) |  |
|  | Not Available | 0 ( 0.0) | 1 ( 0.7) |  |
| gender (%) | FEMALE | 97 (27.7) | 36 (24.0) | 0.453 |
|  | MALE | 253 (72.3) | 114 (76.0) |  |
| grade (%) | G1 | 42 (12.0) | 19 (12.7) | 0.993 |
|  | G2 | 210 (60.0) | 89 (59.3) |  |
|  | G3 | 84 (24.0) | 35 (23.3) |  |
|  | G4 | 1 ( 0.3) | 1 ( 0.7) |  |
|  | GX | 11 ( 3.1) | 5 ( 3.3) |  |
|  | Not Available | 2 ( 0.6) | 1 ( 0.7) |  |
| stage (%) | Stage I | 20 ( 5.7) | 5 ( 3.3) | 0.36 |
|  | Stage II | 55 (15.7) | 26 (17.3) |  |
|  | Stage III | 68 (19.4) | 22 (14.7) |  |
|  | Stage IV | 207 (59.1) | 97 (64.7) |  |
| T (%) | T1 | 27 ( 7.7) | 7 ( 4.7) | 0.361 |
|  | T2 | 92 (26.3) | 51 (34.0) |  |
|  | T3 | 94 (26.9) | 38 (25.3) |  |
|  | T4 | 130 (37.1) | 50 (33.3) |  |
|  | TX | 7 ( 2.0) | 4 ( 2.7) |  |
| M (%) | M0 | 333 (95.1) | 142 (94.7) | 0.887 |
|  | M1 | 3 ( 0.9) | 2 ( 1.3) |  |
|  | MX | 14 ( 4.0) | 6 ( 4.0) |  |
| N (%) | N0 | 169 (48.3) | 72 (48.0) | 0.49 |
|  | N1 | 51 (14.6) | 30 (20.0) |  |
|  | N2 | 111 (31.7) | 41 (27.3) |  |
|  | N3 | 6 ( 1.7) | 1 ( 0.7) |  |
|  | NX | 13 ( 3.7) | 6 ( 4.0) |  |

Table S1 The clinical characteristics of the HNSCC samples in the training and testing sets
