# Supplementary figures and images for: Identification of Prognostic Markers for Head and NeckSquamous Cell Carcinoma Based on Glycolysis-Related Genes
Source: Evid Based Complement Alternat Med. 2022 Jul 7;2022:2762595. doi: 10.1155/2022/2762595 (PMC9283050; doi:10.1155/2022/2762595)

grade

$p = 0.61$

riskScore

3

2

1

G1

G2

G3

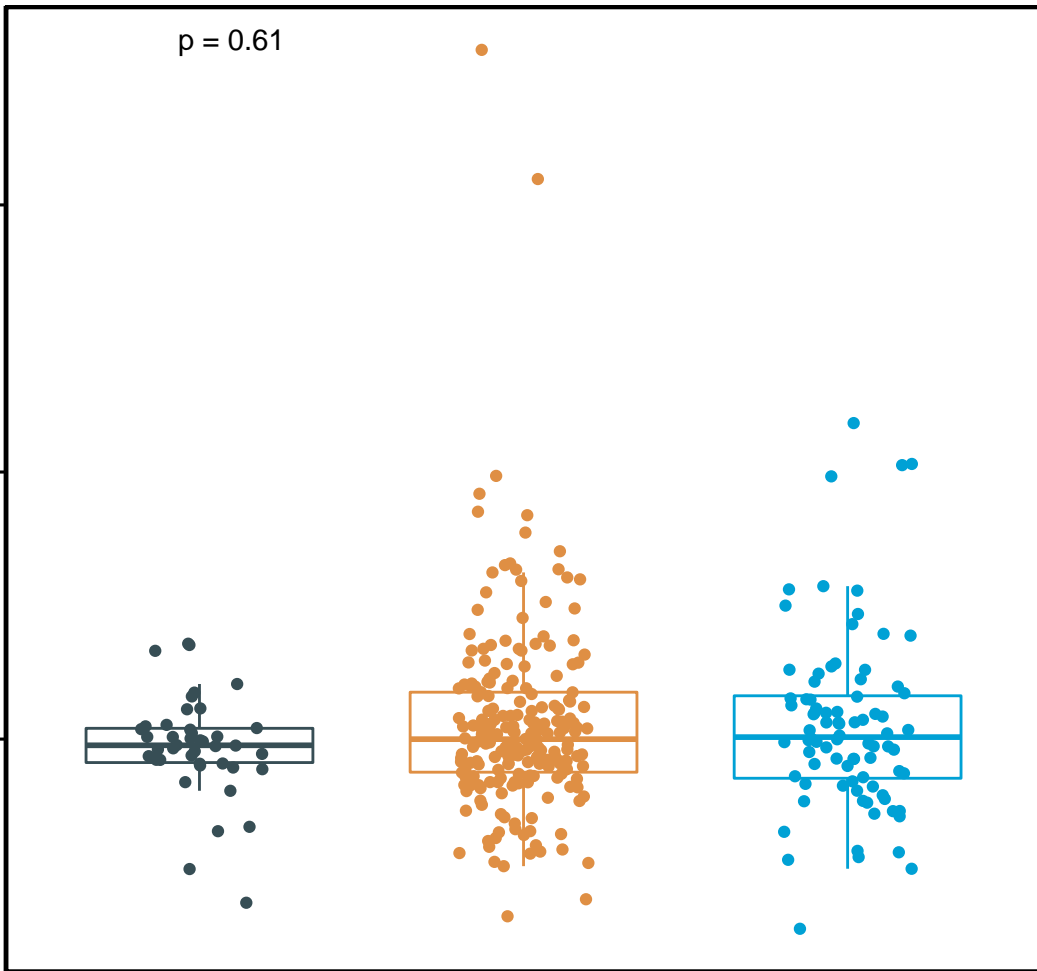

Supplement: Supplementary Materials — Table S1: the clinical characteristics of the HNSCC samples in the training and testing sets. Table S2: a total of 505 DEGs between the HNSCC and normal samples. Table S3: 288 glycolysis-related genes. Figure S1: the correlation between the risk score and clinicopathological characteristics. [file 2762595.f1.zip › 2762595.f1/FigureSupplement 1-A2. grade_riskScore.pdf]
